# Pairing of single-cell RNA analysis and T cell antigen receptor profiling indicates breakdown of T cell tolerance checkpoints in atherosclerosis

**DOI:** 10.1038/s44161-023-00218-w

**Published:** 2023-02-23

**Authors:** Zhihua Wang, Xi Zhang, Shu Lu, Chuankai Zhang, Zhe Ma, Rui Su, Yuanfang Li, Ting Sun, Yutao Li, Mingyang Hong, Xinyi Deng, Mohammad Rafiee Monjezi, Michael Hristov, Sabine Steffens, Donato Santovito, Klaus Dornmair, Klaus Ley, Christian Weber, Sarajo K. Mohanta, Andreas J. R. Habenicht, Changjun Yin

**Affiliations:** 1 Division of Vascular Surgery, The First Affiliated Hospital of Sun Yat-sen University, Guangzhou, China.; 2 Institute for Cardiovascular Prevention (IPEK), Ludwig-Maximilians-University, Munich, Germany.; 3 Institute of Precision Medicine, The First Affiliated Hospital of Sun Yat-sen University, Guangzhou, China.; 4 Department of Oncology, The First Affiliated Hospital of Sun Yat-sen University, Guangzhou, China.; 5 German Center for Cardiovascular Research (DZHK), partner site Munich Heart Alliance, Munich, Germany.; 6 Institute for Genetic and Biomedical Research (IRGB), Unit of Milan, National Research Council, Milan, Italy.; 7 Munich Cluster of Systems Neurology (SyNergy), Ludwig-Maximilians-University, Munich, Germany.; 8 Institute of Clinical Neuroimmunology, University Hospital, Ludwig-Maximilians-University, Munich, Germany.; 9 Immunology Center of Georgia (IMMCG), Augusta University, Augusta, GA, USA.; 10 Cardiovascular Research Institute Maastricht (CARIM), Maastricht University, Maastricht, the Netherlands.; 11 These authors contributed equally: Zhihua Wang, Xi Zhang, Shu Lu, Andreas J. R. Habenicht, Changjun Yin.

## Abstract

Atherosclerotic plaques form in the inner layer of arteries triggering heart attacks and strokes. Although T cells have been detected in atherosclerosis, tolerance dysfunction as a disease driver remains unexplored. Here we examine tolerance checkpoints in atherosclerotic plaques, artery tertiary lymphoid organs and lymph nodes in mice burdened by advanced atherosclerosis, via single-cell RNA sequencing paired with T cell antigen receptor sequencing. Complex patterns of deteriorating peripheral T cell tolerance were observed being most pronounced in plaques followed by artery tertiary lymphoid organs, lymph nodes and blood. Affected checkpoints included clonal expansion of CD4^+^, CD8^+^ and regulatory T cells; aberrant tolerance-regulating transcripts of clonally expanded T cells; T cell exhaustion; T_reg_–TH_17_ T cell conversion; and dysfunctional antigen presentation. Moreover, single-cell RNA-sequencing profiles of human plaques revealed that the CD8^+^ T cell tolerance dysfunction observed in mouse plaques was shared in human coronary and carotid artery plaques. Thus, our data support the concept of atherosclerosis as a bona fide T cell autoimmune disease targeting the arterial wall.

Atherosclerosis is a prototypical chronic inflammatory disease of arteries. Its pathological hallmark is the atherosclerotic plaque in the inner layer of arteries. When plaques rupture, they cause heart attacks or strokes^1–3^. Major innate immune cells have been identified to participate in atherosclerosis. In addition, diverse T cell subtypes including CD8^+^ T cells and CD4^+^ regulatory T (T_reg_) cells promote or attenuate the disease in mice^2,4–6^. However, central questions of T cell immunity in atherosclerosis remain unanswered: in particular, it is not known, whether atherosclerosis-associated T cell responses are carried out in the circulation, in secondary lymphoid organs (SLOs), intima plaques and/or adventitial artery tertiary lymphoid organs (ATLOs)^7,8^. A further elusive although critical issue of atherosclerosis pathogenesis is whether autoimmune T cells are generated and whether they may participate in plaque progression. Recent studies have shown that T cells derived from T_reg_ T cells (exT_reg_ cells) harvested from mice immunized with an ApoB peptide, but not from a control peptide, increase atherosclerotic lesion size and plasma interferon (IFN)-γ levels^5^. Although elimination of autoimmune T cells at central tolerance checkpoints in the thymus contributes to the peripheral T cell repertoire, some thymic autoimmune T cells escape deletion and emigrate into the periphery^9,10^. Here, they form a substantial proportion of the peripheral naїve mature T cell repertoire under physiological conditions^11^. However, while these autoimmune T cells are silenced by multiple strictly regulated mechanisms at various peripheral tolerance checkpoints under physiological conditions, some of them are susceptible to becoming disease-promoting autoimmune T cells under conditions of dysfunctional peripheral tolerance control^12^. Breakdown of these tolerance checkpoints has been shown to occur in unresolved inflammatory diseases, where they can trigger devastating autoimmune diseases, including in multiple sclerosis^13^, type 1 diabetes^14^, rheumatoid arthritis^15^ and psoriasis^16^. However, our comprehension of peripheral tolerance in atherosclerosis remains inadequate.

Here we applied paired 5′ single-cell RNA-sequencing (scRNA-seq) analyses with single-cell T cell antigen receptor (scTCR-seq) profiling using aged *Apoe*^−/−^ mice with advanced atherosclerosis as a model^7,17,18^. This approach allowed us to identify key tissues involved in atherosclerosis-related T cell responses, identify critical T cell subtypes and to examine whether tolerance checkpoints may be compromised. We focused on T cells and myeloid cells in plaques, ATLOs and aorta-draining renal lymph nodes (RLNs). Moreover, integration analyses of scRNA-seq profiles of human plaques indicated that major similarities of CD8^+^ T cell tolerance-related transcriptomes in mice were shared in human coronary and carotid arteries.

## Results

### Transcript maps of T cells and myeloid cells in atherosclerosis

**Extended Data Fig. 1 |:**
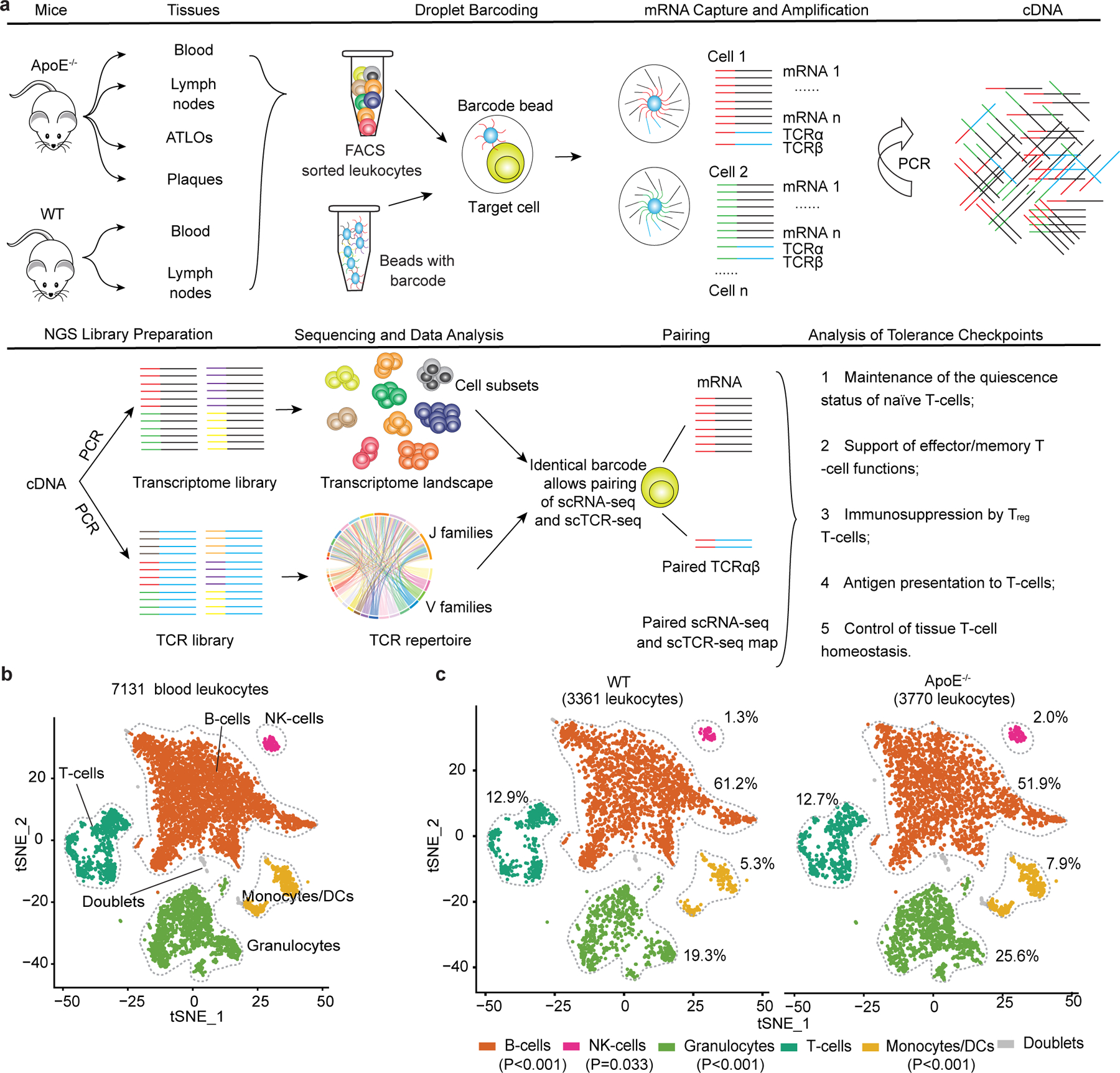
Experimental approach to pairing scRNA-seq analyses with scTCR-seq profiling and blood leukocyte maps of WT and *Apoe*^*−/−*^ mice. **a**, Leukocytes from blood, lymph nodes, ATLOs, and plaques from aged *Apoe*^−*/*−^ and WT mice were purified by FACS sorting and barcoded using the Chromium platform (10x Genomics) for scRNA-seq and scTCR-seq as described in methods. **b**, 7131 blood leukocytes from WT and *Apoe*^−*/*−^ mice were FACS-purified and analyzed by scRNA-seq. 5 major blood immune cell subgroups were defined as T cells, B-cells, monocytes/DCs, NK-cells and granulocytes based on their top 2000 highly expressed genes and graphed as tSNE plots; gray dots represent doublets. **c**, Blood leukocyte subgroup distribution in tSNE plots of WT *vs Apoe*^−*/*−^ mice. The percentage of cells in each subgroup is indicated. Chi-square test followed by Benjamini-Hochberg correction was used to analyze differences.

**Extended Data Fig. 2 |:**
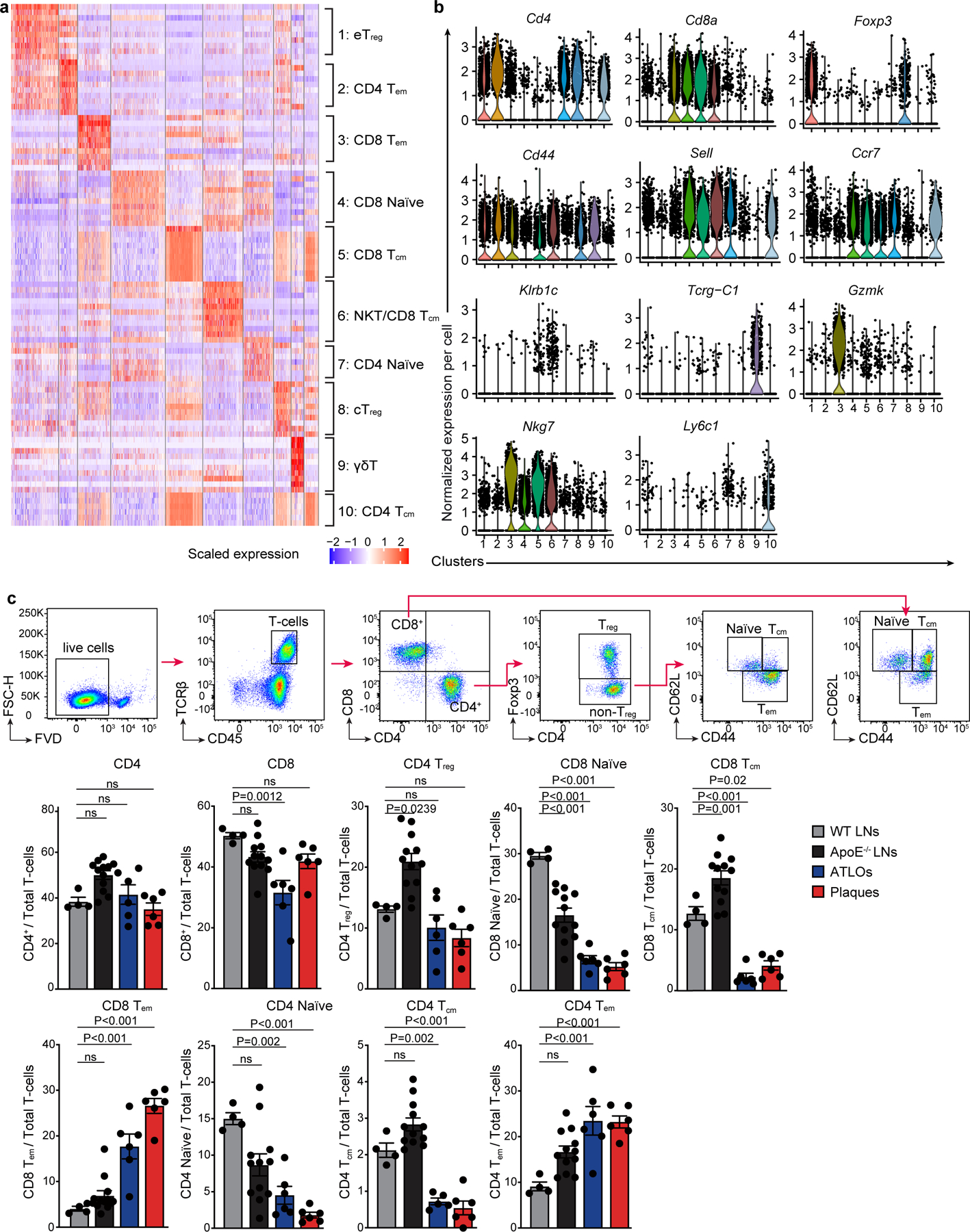
Characterization of T cell subsets by scRNA-seq and FACS in LNs and the diseased arterial wall. **a**, Heatmap shows the top 10 DEGs in each T cell subtype. **b**, Marker combinations define 10 T cell subtypes in Violin plots. *Foxp3* for T_reg_ T cells, *Cd44* for effector/memory T cells, *Sell* (L-selectin or CD62L) and *Ccr7* for naïve T cells, *Klrb1c* (Killer cell lectin-like receptor) for natural killer T- (NKT) cells, *Tcrg-C1* (TCRγ constant region) for γδ T cells, and *Gzmk* (Granzyme K) for CD8 effector T cells. Subset 1: CD4 effector regulatory (eT_reg_) T cells (*Cd4*^+^*Foxp3*^+^*Cd44*^+^*Sell*^−^*Ccr*7^low^); subset 2: CD4 T_em_ T cells (*Cd4*^+^*Foxp3*^−^*Cd44*^+^*Sell*^−^*Ccr7*^low^); subset 3: CD8+ T_em_ T cells (*Cd8a*^+^*Cd44*^+^*Sell*^−^*Ccr7*^low^); subset 4: CD8 naïve T cells (*Cd8a*^+^*Cd44*^−^*Sell*^+^*Ccr7*^high^); subset 5: CD8+ T_cm_ T cells (*Cd8a*^+^*Cd44*^+^*Sell*^+^*Ccr7*^int^); subset 6: NKT/CD8 T_cm_ T cells (*Cd8a*^+^*Cd44*^+^*Sell*^+^*Ccr7*^int^*Klrb1c*^+^); subset 7: CD4 naïve T cells (*Cd4*^+^*Cd44*^−^*Sell*^+^*Ccr7*^high^*Ly6c1*^−^); subset 8: CD4 central T_reg_ (cT_reg_) T cells (*Cd4*^+^*Foxp3*^+^*Ccr7*^low^); subset 9: γδ T cells (*Cd4*^−^*Cd8a*^−^*Tcrg-C1*^+^*Trdc*^+^); subset 10: CD4 T_cm_ T cells (*Cd4*^+^*Cd44*^+^*Sell*^+^*Ccr7*^high^*Ly6c1*^*+*^); each dot represents one cell. **c**, The percentages of T cell subtypes in LNs, ATLOs and plaques of aged WT and *Apoe*^*−/−*^ mice were determined by FACS. Protein marker combinations were used to define T cell subtypes: CD4 naïve T cells (CD4^+^CD44^−^CD62L^+^), CD8 naïve T cells (CD8^+^CD44^−^CD62L^+^); CD4 T_reg_ (CD4^+^Foxp3^+^) T cells, CD8 T_cm_ (CD8^+^CD44^+^CD62L^+^) T cells, CD8 T_em_ (CD8^+^CD44^+^CD62L^−^) T cells, CD4 T_cm_ (CD4^+^Foxp3^−^CD44^+^CD62L^+^) T cells and CD4 T_em_ (CD4^+^Foxp3^−^CD44^+^CD62L^−^) T cells. WT LNs (n = 4), *Apoe*^*−/−*^ LNs (n = 12), ATLOs (n = 6), plaques (n = 6), Data are mean ± s.e.m. One-way ANOVA with Bonferroni *post hoc* test was used to perform statistical analysis.

**Fig. 1 |:**
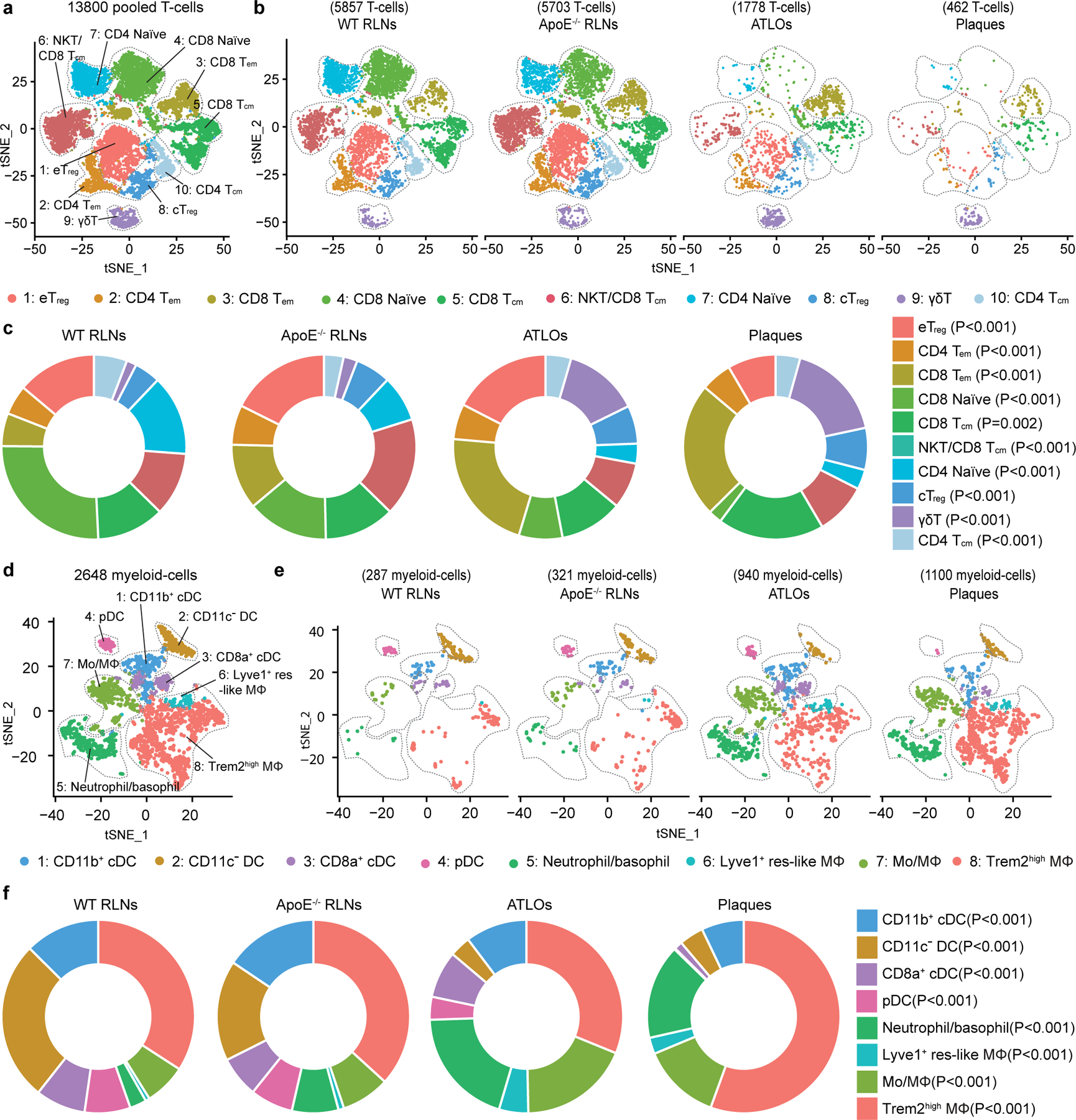
T cell- and myeloid cell-subset-specific maps emerge in the diseased aorta and aorta-draining lymph nodes. **a**, 13,800 T cells were grouped into ten subtypes resulting from *t*-SNE analyses based on their top 2,000 highly expressed genes. **b**, T cell-subset distribution in the *t*-SNE plot of WT RLNs, *Apoe*^−/−^ RLNs, ATLOs and plaques. **c**, Percentages of all T cells of the ten subtypes within each tissue. The size of the colored section represents the percentage of each subset per total T cells. **d**, 2,648 myeloid cells were grouped into eight subtypes resulting from *t*-SNE analyses, including four DC subtypes, three monocyte/macrophage subtypes and one granulocyte subset consisting of basophils and neutrophils. **e**, Myeloid cell-subset distribution in *t*-SNE plots of WT RLNs, *Apoe*^−/−^ RLNs, ATLOs and plaques. **f**, Percentages of eight myeloid cell subsets within each tissue. The size of the colored section represents the percentages for each subset of total myeloid cells. Statistical analysis was performed using Chi-square test with Benjamini–Hochberg correction in **c** and **f**.

To explore peripheral T cell tolerance checkpoints in mouse atherosclerosis, we examined atherosclerotic plaques, ATLOs, aorta-draining RLNs and the circulation using aged *Apoe*^−/−^ mice. As controls, scRNA-seq of blood and RLN T cells of aged and sex-matched wild-type (WT) mice were used^7,8^ (Extended Data Fig. 1a). We identified ten T cell subsets and eight myeloid cell subsets in *Apoe*^−/−^ and WT mice based on their signature gene expression profiles (Fig. 1). Analyses of T cells and myeloid cells in the circulation of *Apoe*^−/−^ versus WT mice showed similar numbers of each of the T cell or myeloid cell subsets (Extended Data Fig. 1b,c) confirming and expanding our previous observation using fluorescence-activated cell sorting (FACS) analyses^7^. Blood T cells, myeloid cells, B cells, natural killer (NK) cells and granulocyte subsets of WT versus *Apoe*^−/−^ mice showed no major numerical differences. We next mapped the territoriality of the T cell and myeloid cell subsets in WT RLNs, *Apoe*^−/−^ RLNs, ATLOs and plaques^19^. Using paired 5′ scRNA-seq with α/β scTCR-seq^20^, we constructed a transcriptome and TCR repertoire atlas comprising 13,800 pooled T cells (Fig. 1a,b). Ten T cell subsets emerged in *t*-distributed stochastic neighbor embedding (*t*-SNE) plots based on their differentially expressed genes (DEGs) and their lead prototypical T cell markers^21,22^ (Fig. 1a,b, Extended Data Fig. 2a,b and Supplementary Table 1). WT RLNs were home to robust naïve CD4^+^ and CD8^+^ T cell pools, central memory (CM) CD44^+^CD62L^+^CD8^+^ T_cm_, effector memory (EM) CD4^+^ T_em_ and T_reg_ cells, and—to a lesser degree—CD8^+^ T_em_ and γδ T cells (Fig. 1b,c). *Apoe*^−/−^ RLNs and ATLOs showed increased numbers of CD4^+^ T_em_ and CD8^+^ T_em_ cells versus WT RLNs (Fig. 1b,c) and ATLOs revealed increased γδ T cells (Fig. 1b,c). Plaques harbored major T cell and myeloid subsets. However, plaque T cell subsets exhibited major aberrant features versus WT RLNs, *Apoe*^−/−^ RLNs and ATLOs. Specifically, plaques contained fewer T_reg_ cells and naïve CD4/CD8^+^ T cells and more CD8^+^ T_cm_, CD8^+^ T_em_ and γδ T cells (Fig. 1b,c). By flow cytometry analysis, we confirmed the increase of CD4^+^ T_em_ and CD8^+^ T_em_ cells in ATLOs when compared to *Apoe*^−/−^ SLOs (Extended Data Fig. 2c). Of note, plaques showed less percentages of naïve CD4^+^/CD8^+^ T cells, and more CD8^+^ T_em_ cells when compared to SLOs (Extended Data Fig. 2c). Thus, a marked disease-associated and tissue-specific T cell composition became apparent.

**Extended Data Fig. 3 |:**
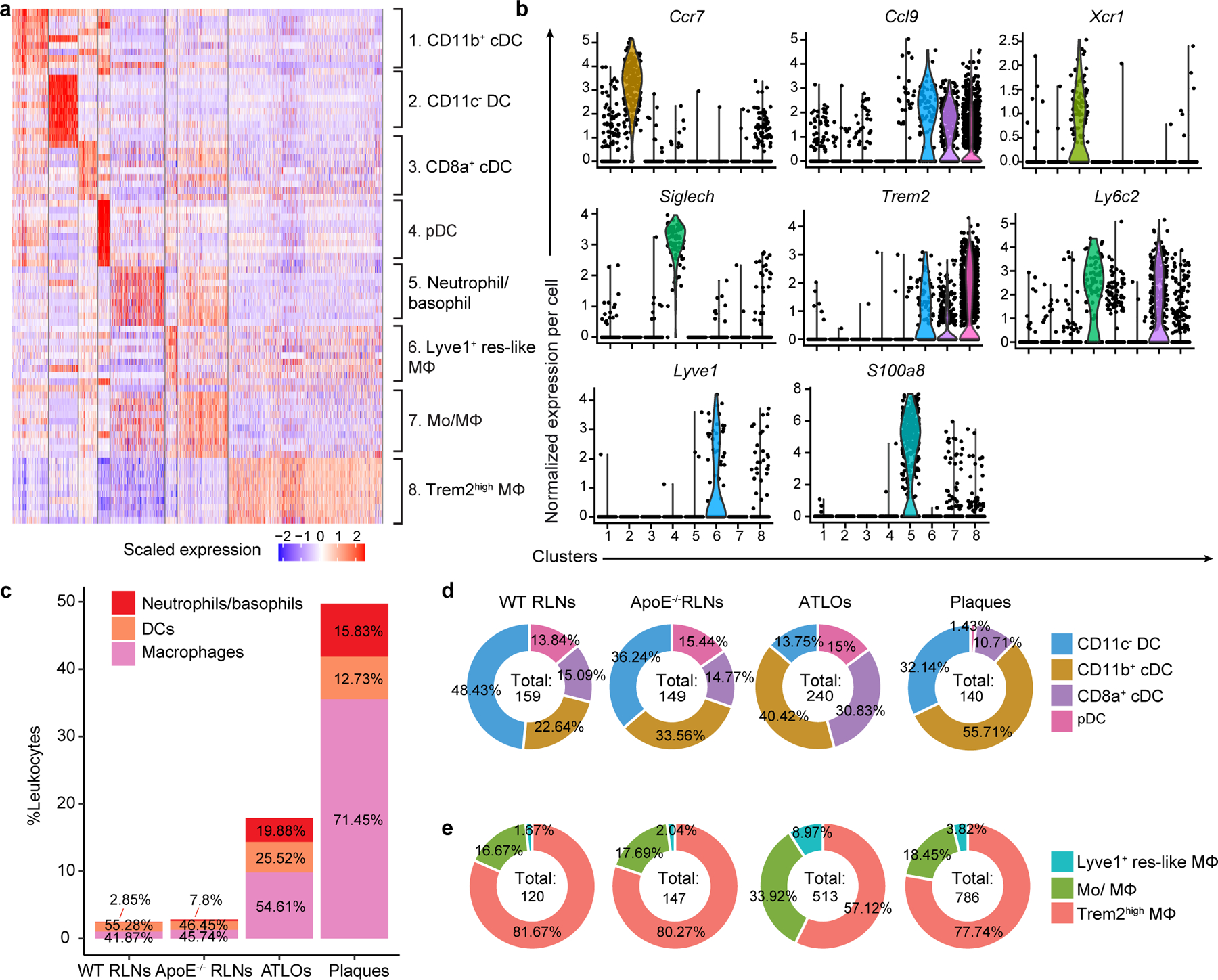
Characterization of myeloid-cell subsets in RLNs and the diseased arterial wall. **a**, Heatmap shows the top 10 DEGs in each myeloid-cell subtype. **b**, Marker combinations used to define 8 myeloid-cell subtypes are listed in Violin plots: subset 1, CD11b^+^ cDC; subset 2, CD11c^−^ DC; subset 3, CD8a^+^ cDC; subset 4, pDC; subset 5, granulocytes including basophils and neutrophils; subset 6, Lyve1^+^ tissue-resident like macrophages; subset 7, monocyte/macrophages; subset 8, Trem2^hi^ macrophages. Each dot represents one cell; *Ccr7* (chemokine receptor involving in homing of T cells and DCs to lymph nodes), *Ccl9* (chemokine or macrophage inflammatory protein-1 gamma attracts DCs); *Xcr1* (chemokine receptor mediating leukocyte migration in response to inflammatory mediators), *SiglecH* (inhibits pDCs inflammatory responses), *Trem2* (mainly expressed by macrophages with potential immunosuppressive activities), *Ly6c2* (a cell surface glycoprotein mainly expressed by monocyte-macrophages and pDCs), *Lyve1* (marker for lymphatic vessels and tissue-resident macrophages with hyaluronan transport activities), *S100a8* (marker for granulocytes with calcium- and zinc-binding activities). **c**, Column graph of numbers of three major myeloid-cell subgroups (Y-axis) in each of four tissues (X-axis). Percentages of basophils/neutrophils, DCs, and macrophages are displayed in each column. **d**, Percentages of each DC subset per total DCs in WT RLNs, *Apoe*^*−/−*^ RLNs, ATLOs and plaques. The total number of DCs analyzed in each tissue is shown in the inner circle. **e**, Percentages of each monocyte/macrophage subset per total macrophages in each tissue. The total number of cells analyzed in each tissue is shown in the inner circle.

Next, we mapped eight myeloid cell subsets including antigen-presenting cells (APCs). Eight myeloid cell subsets were identified according to their lineage marker expression as reported in refs. ^23,24^ (Fig. 1d, Extended Data Fig. 3a,b and Supplementary Table 2). The myeloid cell compartment in plaques and ATLOs expressed as percentage of all leukocytes (49.7% and 17.9%, respectively), exceeded the corresponding compartment in WT and *Apoe*^−/−^ RLNs by a large margin (2.5% and 2.8%, respectively; Extended Data Fig. 3c and Fig. 1e,f). Tolerogenic CD11c^−^ dendritic cell (DCs)^25^ constituted the major DC subset in WT RLNs and *Apoe*^−/−^ RLNs. However, inflammatory conventional DCs (CD11b^+^ cDCs) constituted the major DC subset in ATLOs and plaques (Fig. 1e,f and Extended Data Fig. 3d). Trem2^hi^ macrophages formed the major monocyte/macrophage subtype of WT RLNs, *Apoe*^−/−^ RLNs, ATLOs and plaques (Extended Data Fig. 3e). These data defined a tissue-specific and disease-specific myeloid cell landscape in atherosclerotic mice during aging.

### T cell antigen receptor assembly reveals T cell antigen receptor maps in atherosclerosis

**Extended Data Fig. 4 |:**
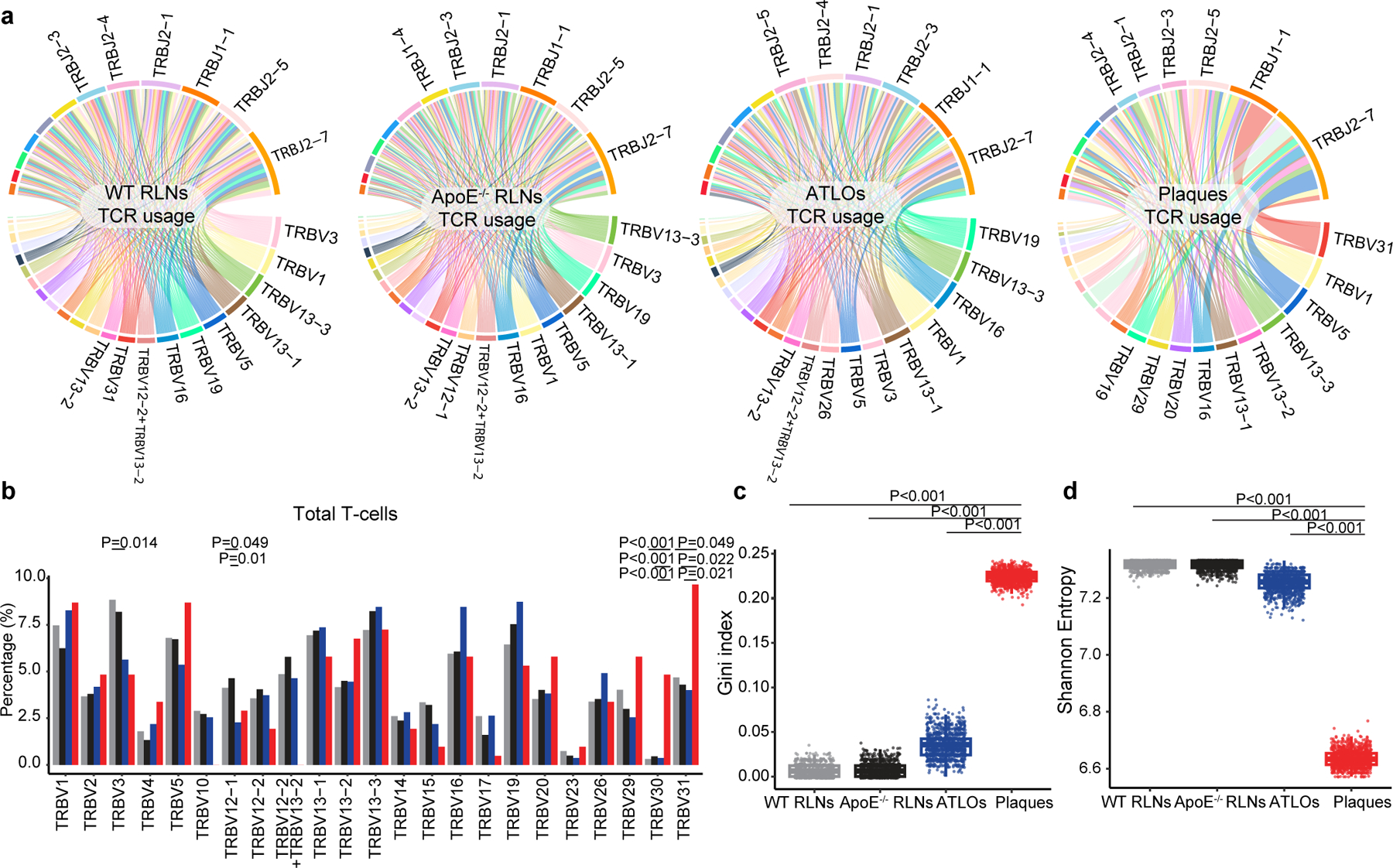
Characterization of the TCR repertoire in RLNs and the diseased arterial wall. **a**, TCRβ chain V- and J-gene usages in WT RLNs, *Apoe*^*−/−*^ RLNs, ATLOs, and plaques. V and J segments are represented by arcs, the size of arcs represents their frequencies. V-J pairings are represented by ribbons. **b**, Distribution of TCRβ V families (TRBV) of total T cells in WT RLNs, *Apoe*^*−/−*^ RLNs, ATLOs, and plaques. Chi-square *post hoc* test with Benjamini-Hochberg correction was performed to analyze difference among tissues in each TRBV families. **c,d**, T cell expansion was calculated by measuring the clonal expansion index (that is Gini index) together with the diversification index (i.e Shannon entropy index) of TCRs. Data from each tissue were randomly down-sampled to the same level (95% of plaque T cells with paired TCRαβ chain, that is 161 cells) and this was repeated 1000 times (**c,d**). The boxplot shows the 25th percentile, median, and 75th percentile values. Each dot represents one time replication. Shapiro-Wilk test was first used to evaluate the data distribution of each group. Data that do not follow normal distribution were analyzed using the Kruskal-Wallis test with Dunn’s non-parametric all-pairs comparison test. The P values were adjusted by Bonferroni correction.

**Fig. 2 |:**
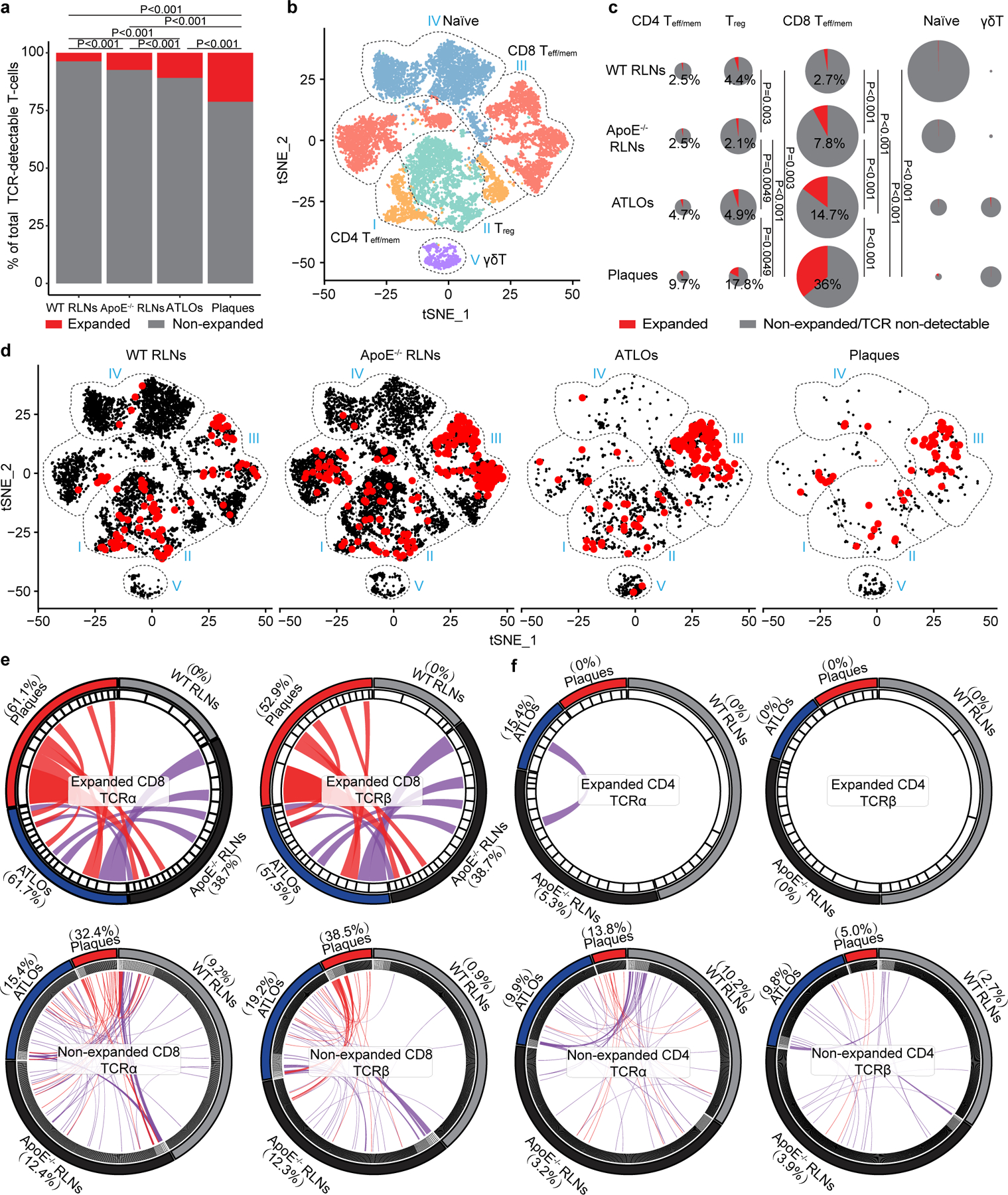
Pairing of scTCR-seq analyses with scRNA-seq profiling reveals tissue-specific TCRα/β maps in advanced mouse atherosclerosis. **a**, Bar plot displays the proportions of clonally expanded (red) versus non-expanded (gray) T cells in different tissues; ≥2 T cells with identical paired TCRα and TCRβ CDR3 sequences are considered as clonally expanded. **b**, *t*-SNE analyses separated a total of 13,800 T cells into ten subtypes, and the cells were grouped into five major subgroups according to their biological functions, including CD4^+^ T_eff/mem_ cells (group I), T_reg_ cells (group II), CD8^+^ T_eff/mem_ cells (group III), naїve T cells (group IV) and γδ T cells (group V). **c**, Pie charts show percentages of clonally expanded versus non-expanded T cells in different groups in different tissues. The sizes of circles represent the relative cell numbers within each tissue. Each circle represents 100%; red indicates the percentages of clonally expanded T cells within each group. **a**,**c**, Chi-square post hoc test with Benjamini–Hochberg correction was used to perform statistical analysis. **d**, Distribution of clonally expanded T cells in *t*-SNE plot. The clonally expanded T cells are highlighted and bolded in red. **e**,**f**, Circos plots show the TCR distribution among four tissues. The number of TCRs of each tissue is represented by arcs displayed in the outer layer of the circle. The size of arcs represents the sample size. Each TCR clone is listed in the inner circle; the size of the inner circle’s segments represents the relative abundance of each clone. Shared TCR clones by two different tissues are represented by ribbons; red represents the shared TCR clones by plaques and other tissues, and purple represents the shared TCR clones by ATLOs, WT RLNs and *Apoe*^−/−^ RLNs. The size of ribbons represents the size of individual shared TCR clones.

We next analyzed the α-chain/β-chain scTCR-seq profiling data to reconstruct their α-chain and β-chain VDJ usage and CDR3 sequences as differential usage of V, D and J has been associated with autoimmune diseases in which compromised central and peripheral tolerance checkpoints have been observed^12^. *Apoe*^−/−^ RLNs, ATLOs and plaques showed differential usages of V family members versus WT RLNs: *Apoe*^−/−^ RLNs were skewed toward TRBV13–3; ATLOs were skewed toward TRBV19 and TRBV16; and plaques were skewed toward TRBV31 and TRBV5 (Extended Data Fig. 4a,b). These data showed that atherosclerosis is associated with differential tissue-specific TCR V family member usages. To get insight into the underlying mechanisms of V family skewing and to determine T cell clonal expansion, we defined expanded T cell clonotypes as ≥2 T cells with identical paired TCRα and TCRβ CDR3 sequences. We found 4% expanded T cells in WT RLNs, 7% in *Apoe*^−/−^ RLNs, 11% in ATLOs and 21% expanded T cells in plaques (Fig. 2a and Extended Data Fig. 4c,d). The paired scRNA-seq/scTCRα/β-seq mapping approach allowed us to determine not only which T cell subsets are clonally expanded in the four tissues but also the transcript signatures of each single expanded T cell in each subtype versus their non-expanded counterparts. For this purpose, we used single-cell barcoding and paired the scRNA-seq with scTCRα/β-seq maps in *t*-SNE projections (Extended Data Fig. 1a). Ten T cell subsets were grouped into five parent T cell subtypes according to their biological functions and their previous exposure to antigens, including both antigen-experienced effector CD4^+^ and memory CD4^+^ T cells (group I, CD4^+^ T_eff/mem_ T cells, which refers to the combined effector T cells and memory T cells), T cells with regulatory functions (group II, including eT_reg_ and cT_reg_ T cells), antigen-experienced effector CD8^+^ T cells and memory CD8^+^ T cells (group III, CD8^+^ T_eff/mem_ T cells), antigen-inexperienced naїve T cells (group IV) and innate-like γδ T cells (group V) (Fig. 2b).

Noticeably, expanded T cell subsets were limited to antigen-experienced CD4^+^ T cells, T_reg_ cells and CD8^+^ T cells (groups I, II and III, respectively; Fig. 2c,d). WT RLNs showed 2.5% and 2.7% expanded CD4^+^ T_eff/mem_ and CD8^+^ T_eff/mem_ T cells, respectively, and 4.4% expanded T_reg_ cells (Fig. 2c,d). However, *Apoe*^−/−^ RLNs and ATLOs showed 2.5% and 4.7% of expanded CD4^+^ T_eff/mem_ T cells, 7.8% and 14.7% of expanded CD8^+^ T_eff/mem_ cells, and 2.1% and 4.9% expanded T_reg_ cells, respectively (Fig. 2c,d). Moreover, plaques showed 9.7% expanded CD4^+^ T_eff/mem_ cells, 36% expanded CD8^+^ T_eff/mem_ T cells, and 17.8% expanded T_reg_ cells (Fig. 2c,d). These data revealed tissue-specific and subset-specific T cell clonal expansion in advanced mouse atherosclerosis, which is particularly pronounced in plaques. These data raised the important possibility that some of these expanded T cell clonotypes have been generated in response to atherosclerosis or arterial wall-derived autoantigens.

Circular plots show the distribution of CDR3 amino acid (aa) sequences. Around 61.1% TCRα and 52.9% TCRβ of expanded CD8^+^ T cells in plaques shared CDR3 aa sequences with expanded CD8^+^ T cells in ATLOs and *Apoe*^−/−^ RLNs but not with expanded CD8^+^ T cells in WT RLNs (Fig. 2e). Curiously, however, expanded CD4^+^ T cells in plaques, did not share CDR3 aa sequences with expanded CD4^+^ T cells in WT or *Apoe*^−/−^ RLNs, or ATLOs (Fig. 2f). Around 2.7% CD4^+^ TCRβ and 0.9% CD8^+^ TCRβ of WT mice shared their CDR3 aa sequences of non-expanded CD4^+^ and CD8^+^ T cells of *Apoe*^−/−^ mice (Fig. 2e,f). These data supported the notion that antigen-experienced CD8^+^ T cells are robustly clonally expanded in plaques, ATLOs and *Apoe*^−/−^ RLNs.

### Naїve peripheral T cell checkpoint in atherosclerosis

**Extended Data Fig. 5 |:**
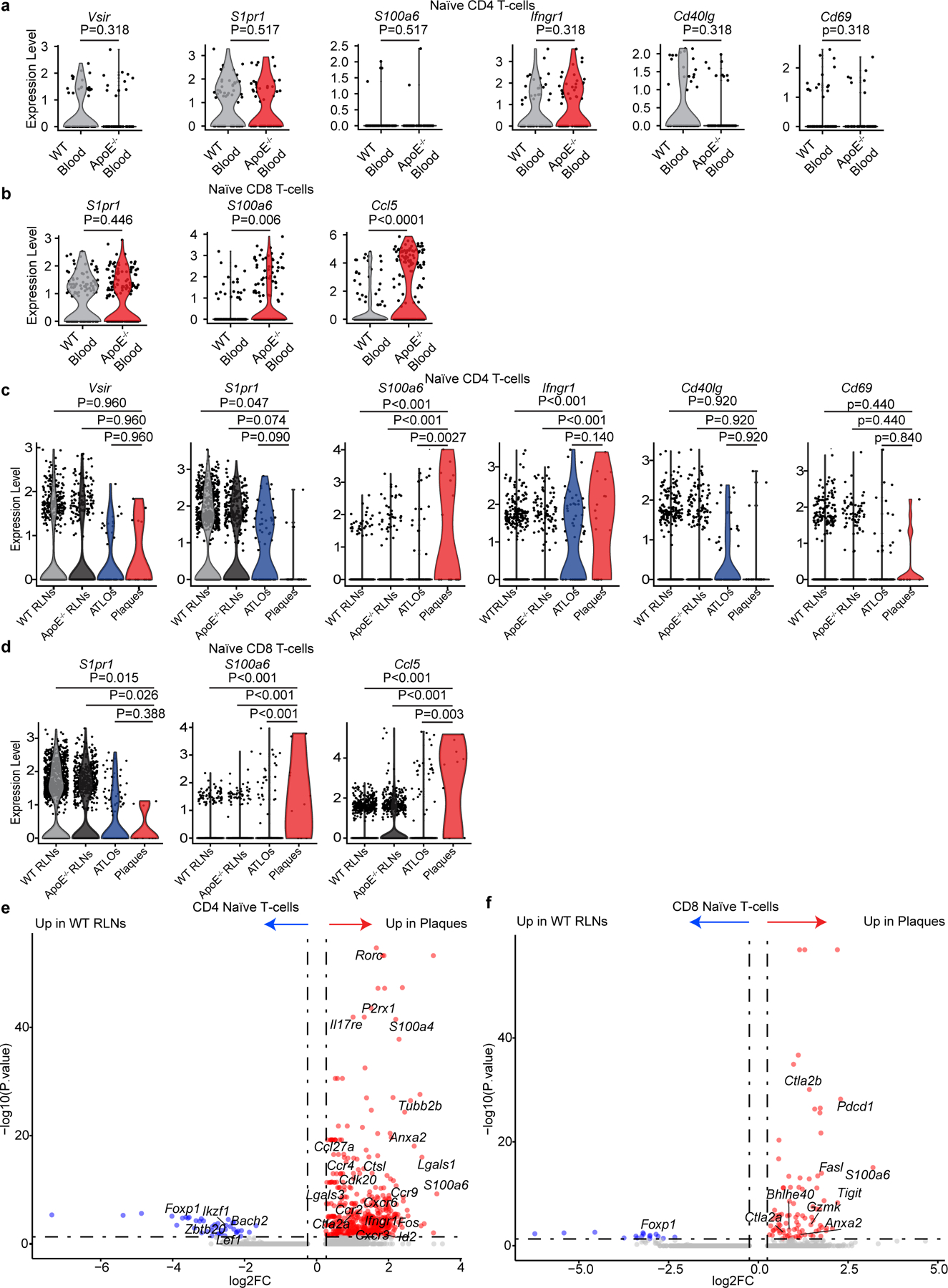
Expression of checkpoint-related transcripts in naïve T cells. **a**, Violin plots of *Vsir*, *S1pr1*, *S100a6*, *Ifngr1*, *Cd40lg*, and *Cd69* transcripts in blood naïve CD4 T cells. **b**, Violin plots of *S1pr1*, *Ccl5*, and *S100a6* transcripts by blood naïve CD8 T cells. Two-sided Wilcoxon rank sum test with Benjamini-Hochberg correction was used to perform statistical analysis (**a,b**). **c**, Violin plots of *Vsir*, *S1pr1*, *S100a6*, *Ifngr1*, *Cd40lg*, and *Cd69* transcripts in naïve CD4 T cells in four tissues. **d**, Violin plots of *S1pr1*, *Ccl5*, and *S100a6* transcripts by tissue naïve CD8 T cells. Each dot represents one single T cell. The difference was calculated by Kruskal-Wallis rank sum test with Dunn’s non-parametric all-pairs comparison test. The P values were adjusted by Benjamini-Hochberg correction (**c**,**d**). **e**, Volcano plot of differentially expressed genes (DEGs) of plaque CD4 naïve T cells *vs* WT RLNs CD4 naïve T cells. **f**, Volcano plot of DEGs of plaque CD8 naïve T cells *vs* WT RLNs of CD8 naïve T cells. Two-sided Wilcoxon-Rank Sum test was used to identify DEGs. Genes with absolute avg_log2FC value >0.25 and adjusted P value <0.05 were considered as significant DEGs. Bonferroni correction was performed to adjust P values (**e**,**f**).

After T cells mature in the thymus, they enter peripheral tissues as naïve (mature) T cells^11^. The body of data described above allowed us to examine five key peripheral tolerance checkpoints of T cell immunity^26^, including maintenance of T cell quiescence; mechanisms to support effector/memory T cell functions; immunosuppression by T_reg_ cells; antigen presentation to T cells; and control of tissue T cell homeostasis. We first examined naïve T cells in the circulation. During their transition to effector and memory T cells, naïve T cells shift from quiescence to an activated/memory-like phenotype by losing expression of inhibitory checkpoint genes including the V-set immunoregulatory receptor (*Vsir*)^26^ and upregulating activation-related transcripts. *Vsir* encodes a prototypic inhibitory checkpoint regulator that prevents activation of naïve CD4^+^ T cells^27^. Therefore, *Vsir* downregulation would be suggestive of an early indicator of tolerance breakdown within the naïve mature T cell repertoire in the circulation. However, the maintenance of the total CD4^+^ naïve T cell pool in the circulation in aged *Apoe*^−/−^ mice was found to be largely intact when compared to WT controls (Extended Data Fig. 5a). Checkpoints controlling naïve CD8^+^ T cell responsiveness are less well understood. We examined the expression of *S1pr1 and* activation-related and cytokine-related transcripts (*S100a6, Ccl5*) in naïve CD8^+^ T cells in the circulation. Interestingly, blood naïve CD8^+^ T cells showed higher levels of *S100a6* and *Ccl5* in *Apoe*^−/−^ versus WT blood (Extended Data Fig. 5b) indicating partially activated naïve CD8^+^ T cells were present in the circulation of aged *Apoe*^−/−^ mice. We next examined these transcripts in naïve CD4^+^ and naïve CD8^+^ cells in WT RLNs, *Apoe*^−/−^ RLNs, ATLOs and plaques. Plaque CD4^+^ naïve T cells showed higher expression of *S100a6* and *Ifngr1* versus WT RLNs (Extended Data Fig. 5c). Plaque naïve CD8^+^ T cells expressed higher levels of *S100a6* and *Ccl5* transcripts (Extended Data Fig. 5d). However, it is noteworthy that there was no apparent difference of the expression of these transcripts between *Apoe*^−/−^ RLNs, ATLOs and WT RLNs (Extended Data Fig. 5d). We next compared all DEGs of CD4^+^ and CD8^+^ naїve T cells in *Apoe*^−/−^ RLNs, ATLOs and plaques versus WT RLNs. Plaque CD4^+^ naïve T cells showed upregulation of activation-regulating (*S100a6*, *Ctsl*, *Ccl27a*, *P2rx1*, *Rorc*, *Il17re*, *Lgasl1*, *Cxcr6*, *Fos*, *Ifngr1*, *Ctla2a*), proliferation-regulating (*Ube2c*, *Cdk20*), migration-regulating (*Ccr2*, *Ccr9*, *Ccr4*) and cellular morphology-regulating (*Tubb2b*, *Anxa2*) transcripts versus WT RLNs (Extended Data Fig. 5e). Plaque CD8^+^ naïve T cells showed upregulation of activation-controlling (*S100a6, Pdcd1*, *Ctla2b*, *Fasl*, *Gzmk*, *Ctla2a*, *Tigit*, *Bhlhe40*) transcripts versus CD8^+^ naïve T cells in WT RLNs (Extended Data Fig. 5f). However, similar relationships between *Apoe*^−/−^ and WT blood in CD4^+^ or CD8^+^ naïve T cells were not observed (Supplementary Table 3). These data support the concept that the maintenance of quiescence of the CD4^+^ and CD8^+^ naїve T cell checkpoint may be compromised with the most pronounced dysfunction in plaques.

### Effector/memory T cell function may be broken

**Extended Data Fig. 6 |:**
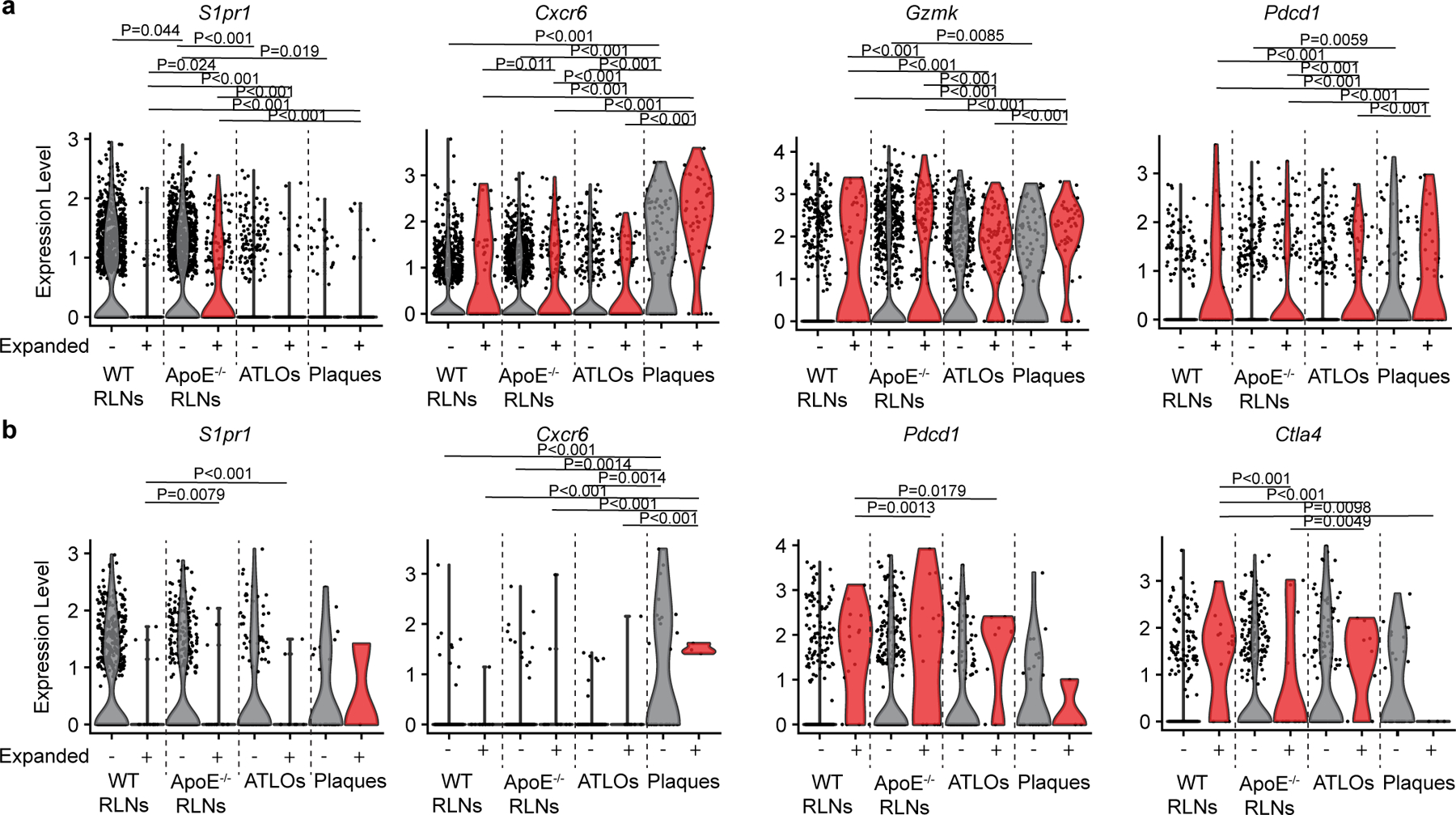
Representative gene expression of checkpoint-related transcripts in CD4 and CD8 T_eff/mem_ T cells. **a**, Violin plots show representative function-relevant gene expression (*S1pr1*, *Cxcr6*, *Gzmk*, and *Pdcd1*) in expanded and non-expanded CD8 T_eff/mem_ T cells of WT RLNs, *Apoe*^*−/−*^ RLNs, ATLOs and plaques. **b**, Violin plots show representative function-associated gene expressions (*S1pr1*, *Cxcr6*, *Pdcd1*, and *Ctla4*) in expanded and non-expanded CD4 T_eff/mem_ T cells of WT RLNs, *Apoe*^*−/−*^ RLNs, ATLOs and plaques. Kruskal-Wallis rank sum test with Dunn’s non-parametric all-pairs comparison test was perform to analyze differences. The P values were adjusted by Benjamini-Hochberg correction (**a**,**b**).

**Fig. 3 |:**
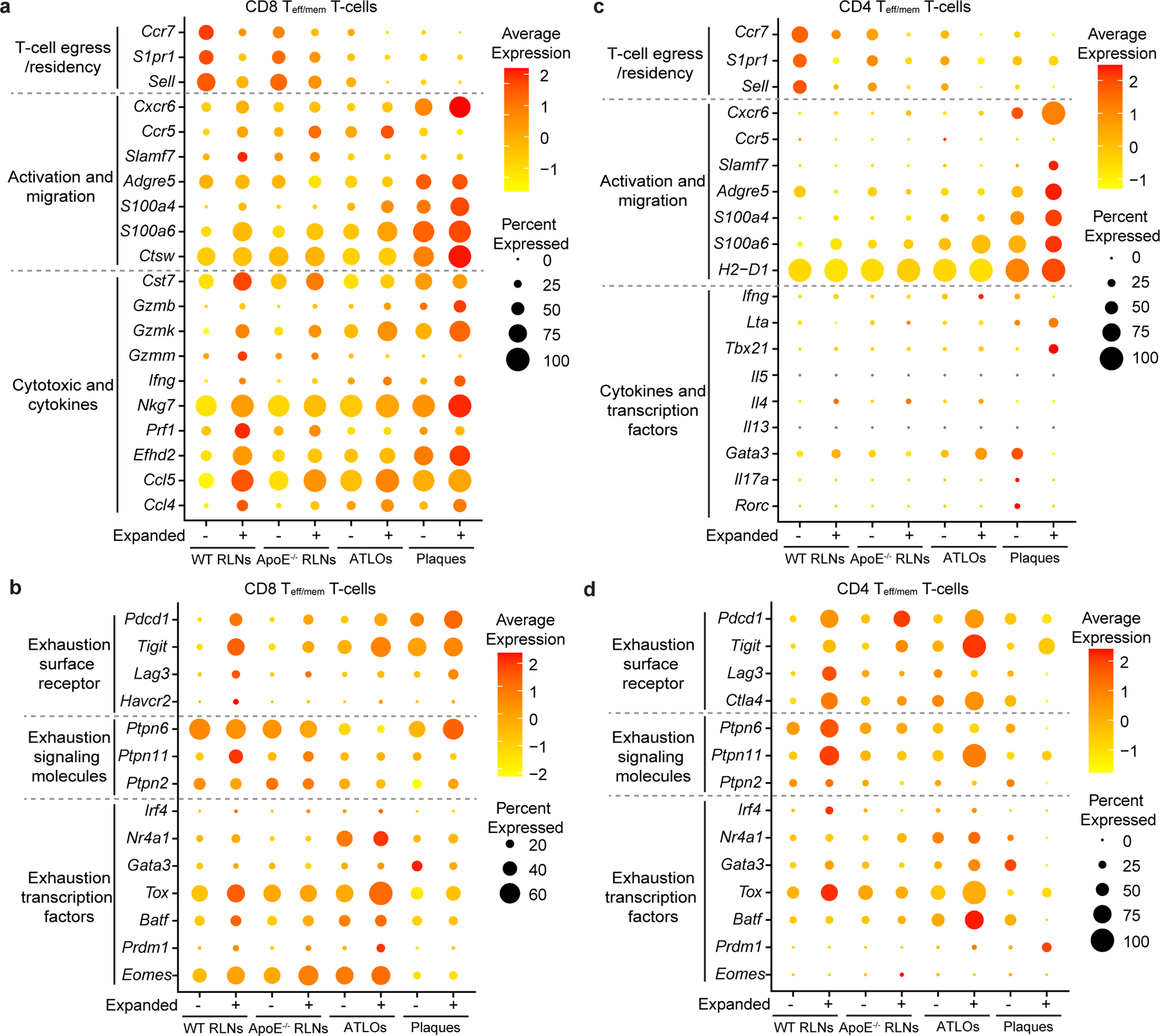
Tolerance phenotypes at the level of transcripts in non-expanded versus expanded plaque CD8+ and CD4+ effector/memory cells. **a**,**b**, Dot plots of gene expression profiles of selected tolerance-associated genes in CD8^+^ T_eff/mem_ cells. Genes related to T cell egress/residency (*Ccr7*, *S1pr1*, *Sell*), activation/migration (*Cxcr6*, *Ccr5*, *Slamf7*, *Adgre5*, *S100a4*, *S100a6*, *Ctsw*), cytotoxicity/cytokines (*Cst7*, *Gzmb*, *Gzmk*, *Gzmm*, *Ifng*, *Nkg7*, *Prf1*, *Efhd2*, *Ccl5*, *Ccl4*); genes related to exhaustion-associated surface receptors (*Pdcd1*, *Tigit*, *Lag3*, *Havcr2*), exhaustion-related signaling molecules (*Ptpn6*, *Ptpn11*, *Ptpn2*) and exhaustion-related transcription factors (*Irf4*, *Nr4a1*, *Gata3*, *Tox*, *Batf*, *Prdm1*, *Eomes*) are listed in **b**. Non-expanded and expanded CD8^+^ T_eff/mem_ cells obtained from WT RLNs, *Apoe*^−/−^ RLNs, ATLOs and plaques are compared. **c**,**d**, Dot plots of gene expression levels of selected function-associated genes in CD4^+^ T_eff/mem_ cells. Genes related to T cell egress/residency (*Ccr7*, *S1pr1*, *Sell*), activation/migration (*Cxcr6*, *Ccr5*, *Slamf7*, *Adgre5*, *S100a4*, *S100a6*, *H2-D1*), transcription factors and cytokines (*Ifng*, *Lta*, *Tbx21*, *Il5*, *Il4*, *Il13*, *Gata3*, *Il17a*, *Rorc*) are listed in **c**; genes related to exhaustion surface receptors (*Pdcd1*, *Tigit*, *Lag3*, *Ctla4*), exhaustion signaling molecules (*Ptpn6*, *Ptpn11*, *Ptpn2*) and exhaustion transcription factors (*Irf4*, *Nr4a1*, *Gata3*, *Tox*, *Batf*, *Prdm1*, *Eomes*) are listed in **d**. Non-expanded and expanded CD4^+^ T_eff/mem_ cells obtained from WT RLNs, *Apoe*^−/−^ RLNs, ATLOs and plaques are compared.

To explore the tolerance checkpoint that controls maintenance of T_eff/mem_ cell function, we compared blood CD4^+^ T_eff/mem_ cells and their CD8^+^ counterparts of WT versus *Apoe*^−/−^ mice. To get detailed insight into the T cell immune response in atherosclerosis, we examined expression of T cell-subtype function-related genes, that is, genes regulating T cell egress/residency, T cell activation/migration/cytotoxicity, T cell cytokine genes and T cell exhaustion-related genes. There was no difference in blood T_eff/mem_ cells in CD4^+^ or CD8^+^ T cells of WT blood versus *Apoe*^−/−^ blood (Supplementary Table 3), indicating that the tolerance checkpoints controlling effector activities and memory of CD4^+^ or CD8^+^ T cell function in the circulation was largely intact. We next phenotyped the clonally expanded versus non-expanded CD8^+^ and CD4^+^ T_eff/mem_ cells (Fig. 3). Non-expanded CD8^+^ and CD4^+^ T_eff/mem_ cells in WT RLNs expressed high levels of transcripts regulating T cell homing/egress/tissue residency (Fig. 3a,c and Extended Data Fig. 6a,b). However, their expanded counterparts expressed lower levels of transcripts regulating T cell egress from lymphoid organs (*S1pr1*), higher expression of activation/cytotoxic/cytokine-related transcripts (*Slamf7*, *S100a6*, *Gzmm*, *Gzmk*, *Nkg7*, *Ccl5*, *Ccl4*, *Ifng*, *Prf1*) and dysregulation of exhaustion-related genes (*Pdcd1*, *Tigit*, *Lag3*, *Havcr2* on CD8^+^*, Ctla4* on CD4^+^) versus their non-expanded counterparts in WT RLNs (Fig. 3). These data indicate that T cell clonal expansion impairs T cell migration, cytokine production and exhaustion functions of both CD4^+^ and CD8^+^ T cells under physiological conditions in WT lymphoid organs as expected^28^. *Apoe*^−/−^ RLNs and ATLOs showed major phenotypic similarities to their WT RLN non-expanded CD8^+^ T cell brethren (Fig. 3a,b). However, *Apoe*^−/−^ RLN-derived and ATLO-derived expanded CD8^+^ and CD4^+^ T_eff/mem_ T cells showed decreased expression of T cell egress-related transcripts (*Ccr7*, *S1pr1*, *Sell*), and increased expression of exhaustion-related genes (*Pdcd1*, *Tigit*, *Ctla4*, *Tox*, *Batf and Eomes*; Fig. 3 and Extended Data Fig. 6a,b). These data suggest that T cell migration, cytokine production and exhaustion functions are markedly dysregulated during T cell clonal expansion in *Apoe*^−/−^ RLNs and ATLOs. Surprisingly, both non-expanded and expanded CD8^+^ T_eff/mem_ cells in plaques showed decreased expression of egress-related transcripts, increased activation/cytokine production and exhaustion-regulating transcripts versus WT RLNs (Fig. 3a,b and Extended Data Fig. 6a). However, *Apoe*^−/−^ RLN-derived and ATLO-derived non-expanded CD4^+^ T_eff/mem_ cells displayed decreased expression of T cell egress-related transcripts but similar expression profiles of activation/migration-related, cytokine-related and exhaustion-related transcripts versus WT cells in RLNs (Fig. 3c,d and Extended Data Fig. 6b). Plaque non-expanded and expanded CD4^+^ T_eff/mem_ cells revealed increased expression of T cell activation/migration/cytokine signature-related transcripts, but surprisingly, decreased expression of exhaustion-related transcripts—unlike CD8^+^ T cells—versus WT T cells (Fig. 3c,d and Extended Data Fig. 6b). These data support the notion that tolerance breakdown in atherosclerosis is largely tissue specific regarding effector and memory functions in mouse advanced atherosclerosis and most pronounced in plaques followed by ATLOs and *Apoe*^−/−^ RLNs.

**Extended Data Fig. 7 |:**
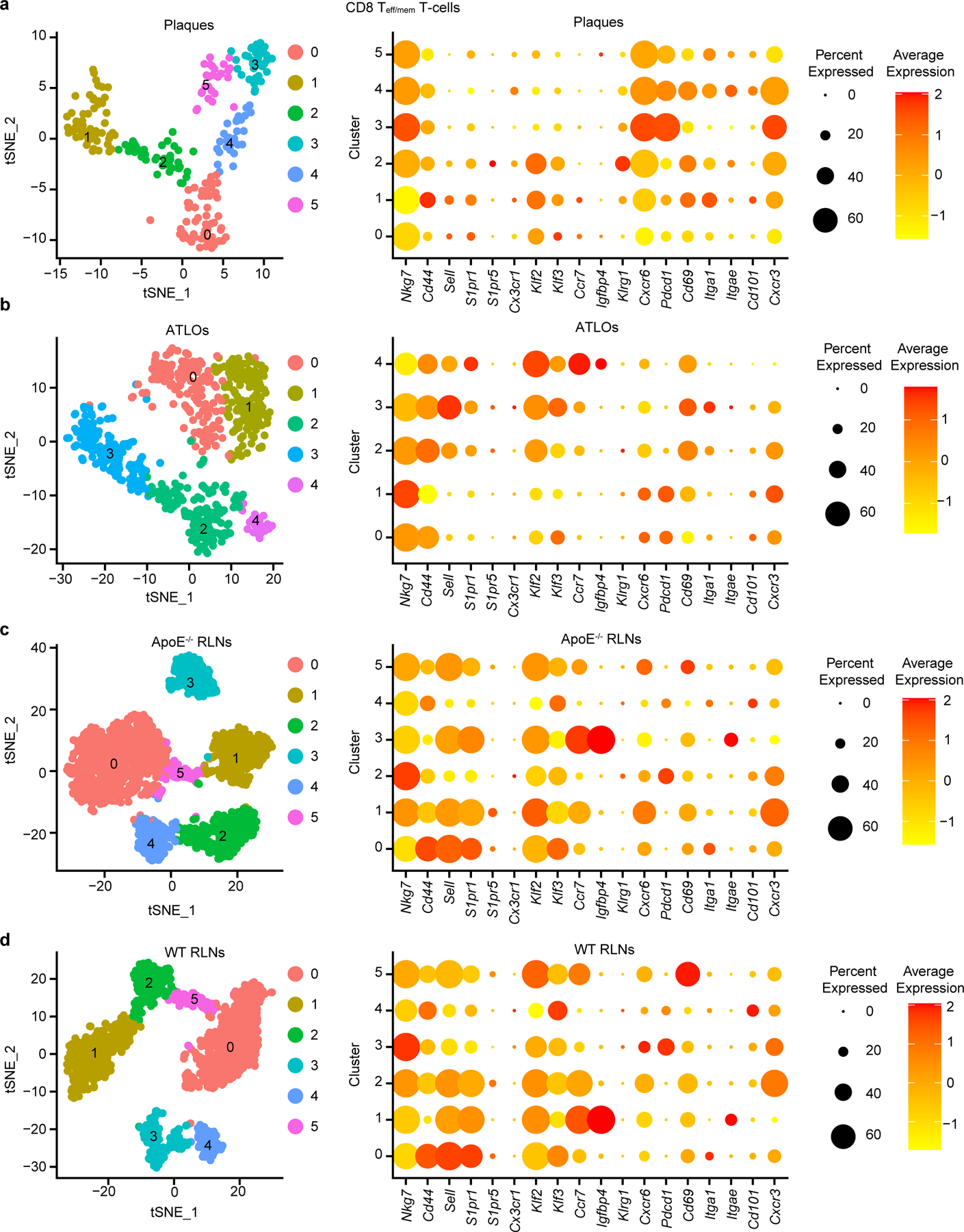
Average expression of genes with similarities to tissue-resident memory (TRM)-like T cells in different CD8 T_eff/mem_ T cells among different tissues. **a**, 237 plaque CD8 T_eff/mem_ T cells were grouped into 6 subtypes resulting from tSNE analyses based on their top 2000 highly expressed genes; Dot-plot shows the average expression of genes associated with T cell egression-, activation-, and TRM-like T cell phenotypes; **b**, 726 ATLO CD8 T_eff/mem_ T cells were grouped into 5 subtypes; **c**, 2339 *Apoe*^*−/−*^ RLNs CD8 T_eff/mem_ T cells were grouped into 6 subtypes; **d**, 1688 WT RLNs CD8 T_eff/mem_ T cells were grouped into 6 subtypes; CD8 T_eff/mem_ T cells were obtained from three independent experiments. Each dot represents one single cell.

We next examined the gene signatures of tissue-resident memory (TRM) T cells^29^ in plaques as TRM T cells have not been identified in atherosclerosis. Three major memory T cell subsets were defined, that is, TRM-like T cell, T_cm_ cells and T_em_ cells. TRM T cells have been described as a non-circulating T cell pool that is thought to permanently reside in tissues, and express tissue-specific gene signatures^29^. To examine TRM cell signatures in each tissue independently, we reclustered all CD8^+^ T_eff/mem_ cells in each tissue (Extended Data Fig. 7). Plaque CD8^+^ memory T cells were divided into six clusters: clusters 0, 1 and 2 expressed higher levels of *Sell* and *S1pr1* when compared to other clusters in plaques, indicating clusters 0, 1 and 2 largely contain CD8^+^ T_cm_ cells (Extended Data Fig. 7a). Cluster 3 expressed higher levels of *Cxcr6 and Pdcd1* but expressed lower levels of TRM-related genes, including *Cd69 and Itgae*, indicating that cluster 3 represents largely exhausted CD8^+^ T_em_ cells (Extended Data Fig. 7a). Gene expression profiles showed that cluster 4 and 5, however, revealed higher expression levels of TRM-type genes and lower expression levels of egression genes including high expression levels of *Itga1*, *Itgae* and *Cd101*, along with low expression levels of *Sell*, *S1pr1*, *S1pr5*, *Klf2, Klf3* and *Ccr7* (Extended Data Fig. 7a). We therefore named clusters 4 and 5 of T cells in plaques as CD8^+^ TRM-like T cells. Using the same strategy, we reclustered CD8^+^ T_eff/mem_ cells in ATLOs, *Apoe*^−/−^ RLNs and WT RLNs (Extended Data Fig. 7b–d) and CD4^+^ T_eff/mem_ cells in all tissues (Supplementary Fig. 1). CD8^+^ or CD4^+^ T_eff/mem_ cells showed typical CD8^+^ or CD4^+^ T_em_ (*Cd44*^+^*Sell*^−^*Ccr7*^−^) and CD8^+^ or CD4^+^ T_cm_ (*Cd44*^+^*Sell*^*+*^*Ccr7*^*+*^) T cell expression profiles (Extended Data Fig. 7b–d and Supplementary Fig. 1). Cluster 3 of CD8^+^ T cells in ATLOs, and cluster 5 of CD8^+^ T cells in WT and *Apoe*^−/−^ RLNs highly expressed *Cd69* but with high levels of *Sell*, *Klf2* and *Klf3* and low levels of other TRM-related markers (Extended Data Fig. 7b–d), indicating that they do not qualify as CD8^+^ TRM T cells. Further work is warranted to reveal the functional impacts of plaque CD8^+^ TRM-like cells in atherosclerosis progression.

### Regulatory T cell dysfunction-related transcripts in plaques

**Fig. 4 |:**
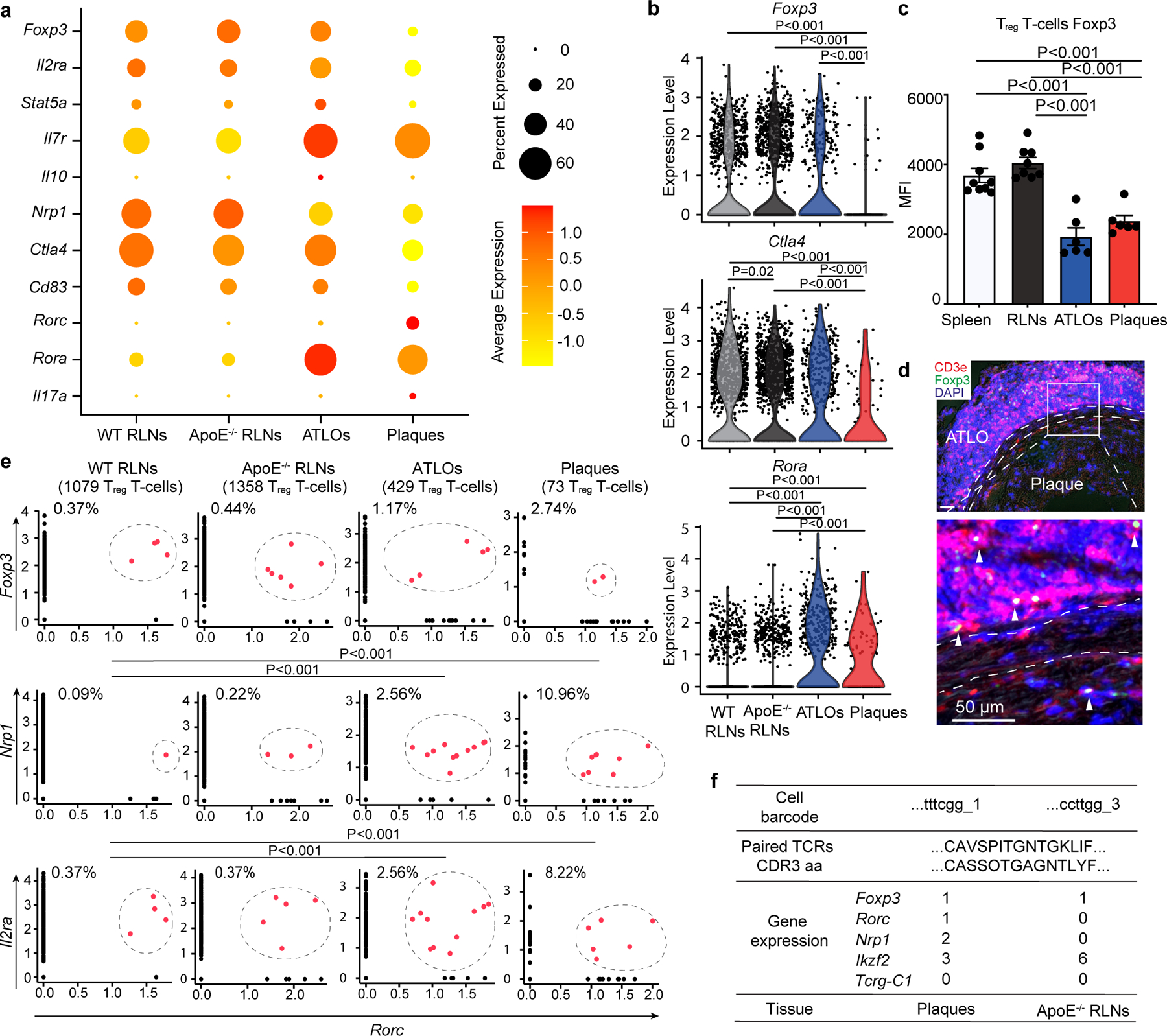
T_reg_–TH_17_ T cell conversion in atherosclerotic plaques. **a**, Dot plots of expression levels of selected function-associated genes in T_reg_ cells. Genes related to IL-2/STAT5 activation (*Foxp3*, *Il2ra*, *Stat5a*), genes highly expressed by effector T_reg_ cells (*Il10*, *Nrp1*, *Ctla4*, *Cd83*) and IL-17-signaling pathway-related genes (*Rorc*, *Rora*, *Il17a*). T_reg_ cells obtained from WT RLNs, *Apoe*^−/−^ RLNs, ATLOs and plaques were compared. **b**, Violin plots show representative gene expression levels associated with T_reg_ cells. Each dot represents one single T_reg_ cell. Kruskal–Wallis rank-sum test with Dunn’s non-parametric all-pairs comparison test. The *P* values were adjusted by Benjamini–Hochberg correction. **c**, Mean fluorescence intensity (MFI) of Foxp3 protein in T_reg_ cells of spleen, RLNs, ATLOs and plaques in aged *Apoe*^−/−^ mice. CD4^+^Foxp3^+^ protein markers were used to define T_reg_ cells. *Apoe*^−/−^ spleens (*n* = 9), *Apoe*^−/−^ RLNs (*n* = 8), ATLOs (*n* = 6), plaques (*n* = 6). Data are the mean ± s.e.m. Statistical analysis was performed using one-way analysis of variance with Bonferroni post hoc test. **d**, Anti-CD3e and anti-Foxp3 were used to stain aortic sections of aged *Apoe*^−/−^ mice. Data are representative of three experiments. **e**, Double-positive cells of T_reg_–TH_17_ converting cells. Coexpression of T_reg_ and TH_17_ cell markers in single T_reg_ cells are labeled in red. Each dot represents a single T_reg_ cell. 1,079 T_reg_ cells in WT RLNs, 1,358 T_reg_ cells in *Apoe*^−/−^ RLNs, 429 T_reg_ cells in ATLOs and 73 T_reg_ cells in plaques were examined. Chi-square test was used to perform statistical analysis. **f**, Two T cells with identical paired TCR sequences. Cell barcode, the CDR3 aa sequences of paired TCR sequences, gene expression and tissue origin are shown.

We next examined T_reg_ cells, which control autoimmunity at peripheral tolerance checkpoints by targeting both innate and adaptive immune cells by multiple mechanisms including high expression of immunosuppressive cytokines and receptors, that is, encoded by *Il10*, *Tgfb1* and *Ctla4* genes^30^. We first delineated T_reg_ cell function-related genes^31^ (Fig. 4a). T_reg_ cells in WT RLNs highly expressed genes related to the maintenance of T_reg_ cells (*Foxp3*, *Il2ra*, *Stat5a*) and their suppressive activities (*Il10*, *Nrp1*, *Ctla4*, *Cd83*), as expected (Fig. 4a,b). Moreover, T_reg_ cells in *Apoe*^−/−^ RLNs and ATLOs expressed similar levels of these genes (Fig. 4a,b). However, T_reg_ cells in plaques expressed lower levels of *Foxp3*, *Il2ra*, *Stat5a*, *Il10*, *Nrp1*, *Ctla4* and *Cd83* versus WT RLNs (Fig. 4a,b). We next determined the Foxp3 protein levels in T_reg_ cells of different tissues in aged *Apoe*^−/−^ mice by FACS. CD4 Foxp3^+^ T cells expressed high levels of Foxp3 protein levels in *Apoe*^−/−^ SLOs. However, ATLO and plaque CD4^+^ Foxp3^+^ T cells expressed significantly lower levels of Foxp3 protein (Fig. 4c), suggesting destabilization and loss of their suppressive functions. By immunofluorescence staining, we observed markedly less Foxp3^+^ T_reg_ cells in plaques when compared to ATLOs (Fig. 4d). These data indicate that T_reg_ cell function is not compromised in *Apoe*^−/−^ SLOs, but that it is in plaques and to a lesser degree in ATLOs. By using various fate reporter labeling technologies and adoptive transfer approaches, the loss of Foxp3 has been reported to facilitate the pathogenic conversion of Foxp3^+^ T_reg_ cells into TH_17_ cells in autoimmune arthritis^15^, and more recently in ApoB-specific T_reg_ cells in atherosclerosis^32^. To determine whether T_reg_–TH_17_ cell conversion may occur in aged WT and *Apoe*^−/−^ mice, we examined expression of TH_17_ T cell-related transcripts in T_reg_ cells. All T_reg_ cells in WT RLNs, *Apoe*^−/−^ RLNs and ATLOs expressed low and similar levels of TH_17_ T cell-related transcripts (*Il17a*, *Rora*, *Rorc*; Fig. 4a). Plaque T_reg_ cells expressed higher TH_17_-related transcripts versus all other tissues (Fig. 4a). These data were consistent with the possibility that plaque T_reg_ cells convert to TH_17_-like T cells specifically and selectively in advanced plaques. To obtain direct evidence to support the T_reg_–TH_17_ T cell conversion in vivo, we searched for T cells coexpressing both T_reg_ and TH_17_ cell markers. Coexpression of *Foxp3* and *Rorc*, *Nrp1* and *Rorc*, and *Il2ra* and *Rorc* in T cells provides evidence at the level of their transcript profiles for T_reg_–TH_17_ cell conversion in aged WT and *Apoe*^−/−^ mice.

We observed that few T cells coexpressed the transcription factors encoded by the *Foxp3* and the *Rorc* genes: 0.37% (4/1,079) T_reg_ cells in WT RLNs, 0.44% (6/1,358) T_reg_ cells in *Apoe*^−/−^ RLNs, 1.17% (5/429) T_reg_ cells in ATLOs and 2.74% (2/73) T_reg_ cells in plaques (Fig. 4e). However, T_reg_ cells revealed low expression levels of *Foxp3* in plaques in addition to their cell surface markers *Nrp1* and *Il2ra/*CD25, which are prototypically expressed by T_reg_ cells indicating that some T_reg_ cells may have entered the T_reg_–TH_17_ T cell conversion pathway. Approximately 0.09% (1/1,079) of T_reg_ cells in WT RLNs, 0.22% (3/1,358) of T_reg_ cells in *Apoe*^−/−^ RLNs, 2.56% (11/429) of T_reg_ cells in ATLOs and 10.96% (8/73) of T_reg_ cells in plaques were double positive for *Nrp1* and *Rorc* (Fig. 4e); 0.37% (4/1,079) of T_reg_ cells in WT RLNs, 0.37% (5/1,358) of T_reg_ cells in *Apoe*^−/−^ RLNs, 2.56% (11/429) of T_reg_ cells in ATLOs and 8.22% (6/73) of T_reg_ cells in plaques were double positive for *Il2ra* and *Rorc* (Fig. 4e). These data showed marked T_reg_–TH_17_ conversion in plaques, and to a lesser degree in ATLOs versus WT RLNs and *Apoe*^−/−^ RLNs. Interestingly, we found two T cells carrying identical paired TCRαβ chains (so-called twin-like T cells), one of which was a *Foxp3*^+^*Rorc*^−^ bona fide T_reg_ cell obtained from *Apoe*^−/−^ RLNs, while its brethren was *Foxp3*^+^*Rorc*^+^ and thus this T cell represents a T_reg_ cell in the process of being converted to a TH_17_ cell in plaques (Fig. 4f). That two daughters of the identical T_reg_ cell with two different fates—normal T_reg_ cells in *Apoe*^−/−^ RLNs versus converting T_reg_–TH_17_ in plaques—have been defined further supports the notion of T_reg_–TH_17_ conversion in atherosclerosis and here in plaques. When taken together, these data are consistent with the interpretation that three distinct mechanisms may contribute to T_reg_ cell tolerance checkpoint dysfunction in ATLOs and plaques when compared to WT RLNs, that is, (1) loss of T_reg_ cell number; (2) loss of immunosuppressive-related transcripts; and (3) T_reg_–TH_17_ conversion.

### Antigen presentation to T cells is compromised

**Extended Data Fig. 8 |:**
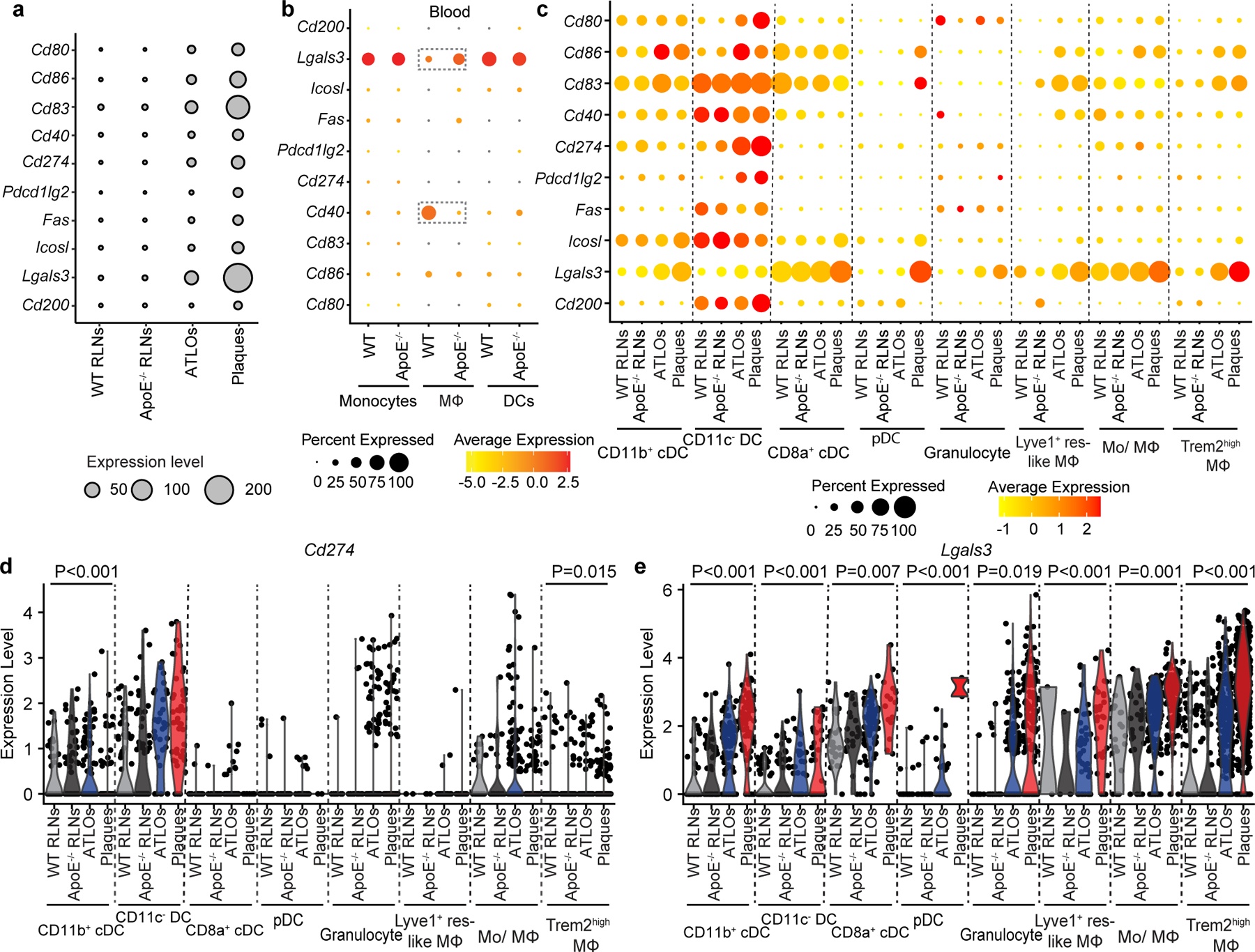
Dysregulation of costimulatory- and checkpoint inhibitor-related transcripts in APCs in ATLOs and plaques but not in RLNs of WT or *Apoe*^*−/−*^ mice. **a**, Myeloid-cell-derived costimulatory genes (*Cd80*, *Cd86*, *Cd83*, *Cd40*) and checkpoint inhibitor genes (*Cd274*/PD-L1, *Pdcd1lg2*/PD-L2, *Fas*, *Icosl*, *Lgals3*, *Cd200*) in CD45^+^ leukocytes in four tissues. Expression was normalized: Expression = (Average gene expression per cell type)*(Percentage of cell type per CD45^+^ leukocytes). **b**, Myeloid-cell-derived checkpoint inhibitor genes (*Cd274*/PD-L1, *Pdcd1lg2*/PD-L2, *Fas*, *Icosl*, *Lgals3*, *Cd200*) and costimulatory genes (*Cd80*, *Cd86*, *Cd83*, *Cd40*) per CD45^+^ leukocytes between WT blood *vs Apoe*^*−/−*^ blood. **c**, The average expression of costimulatory genes and checkpoint inhibitor genes by different myeloid-cell subsets. **d,e**, Violin plots show *Cd274* and *Lgals3* gene expression by different myeloid-cell subsets. Each dot represents one single cell. Two-sided Kruskal-Wallis rank sum test was used to analyze the differences among groups.

T cell checkpoint inhibitors are known to be expressed by APCs, including DCs and macrophages^33,34^. In view of the major role of APCs in the regulation of peripheral tolerance, we examined the expression by myeloid cells including DCs/macrophages/granulocytes of their costimulatory genes (*Cd80*, *Cd86*, *Cd83*, *Cd40*) and checkpoint inhibitor genes (*Cd274/*PD-L1, *Pdcd1lg2/*PD-L2, *Fas*, *Icosl*, *Lgals3*, *Cd200*; Extended Data Fig. 8a). We observed major overlaps between blood monocytes and DCs in gene expression signatures between WT and *Apoe*^−/−^ blood (Extended Data Fig. 8b), indicating that most genes regulating myeloid cell tolerance checkpoints in blood APCs are largely unchanged. Moreover, *Apoe*^−/−^ RLNs also showed major similarities on expression of costimulatory genes and checkpoint inhibitor genes versus WT RLNs (Extended Data Fig. 8a). However, ATLOs and plaques revealed increased expression of costimulatory and checkpoint inhibitor-related genes when compared to WT RLNs (Extended Data Fig. 8a). These data indicated dysregulation of the balance between costimulatory and inhibitory activities of APCs in ATLOs and plaques but not in the circulation or in RLNs of either genotype consistent with the important possibility that tolerance is regulated in the periphery and especially in the diseased arterial wall. We next examined the patterns of costimulatory-related and inhibitory-related transcripts in different myeloid cell subsets (Extended Data Fig. 8c). WT RLN CD11c^−^ DCs expressed high levels of *Cd83*, *Cd40*, *Cd274/*PD-L1, *Fas*, *Icosl* and *Cd200* indicative of a tolerogenic DC phenotype^33^ (Extended Data Fig. 8c). Yet, CD11c^−^ DCs in plaques, but not their brethren in *Apoe*^−/−^ RLNs and ATLOs, expressed higher levels of costimulatory genes (*Cd80*, *Cd86*) and checkpoint inhibitor genes (*Cd274/*PD-L1, *Pdcd1lg2*) when compared to WT CD11c^−^ DCs (Extended Data Fig. 8c). These data indicated dysfunction of tolerogenic DCs in plaques, but not in *Apoe*^−/−^ RLNs or ATLOs. Moreover, Trem2^hi^ macrophages had higher expression levels of *Cd86* and *Lgals3* in ATLOs and plaques versus WT RLNs (Extended Data Fig. 8c), indicating that these cells increase their costimulatory and checkpoint inhibitory functions in ATLOs and plaques. We next examined single-cell expression of checkpoint inhibitor-related transcripts *Cd274* and *Lgals3*, that is, genes that limit T cell functions by binding to T cell surface programmed death-1 (PD-1) and lymphocyte activation gene-3 (LAG-3), respectively. WT RLNs CD11b^+^ cDCs and CD11c^−^ DCs selectively expressed *Cd274* (Extended Data Fig. 8d). WT RLNs CD11b^+^ cDCs, CD11c^−^ DCs, CD8α^+^ cDCs, Lyve1^+^ macrophages and monocyte/macrophages expressed *Lgals3* (Extended Data Fig. 8e). *Apoe*^−/−^ RLNs, ATLOs and plaques showed tissue-specific and cell-subset-specific dysregulation of these two genes, with the most pronounced phenotype in plaques (Extended Data Fig. 8e), indicating that this key tolerance checkpoint controlling T cell immunity is compromised in myeloid cells.

**Extended Data Fig. 9 |:**
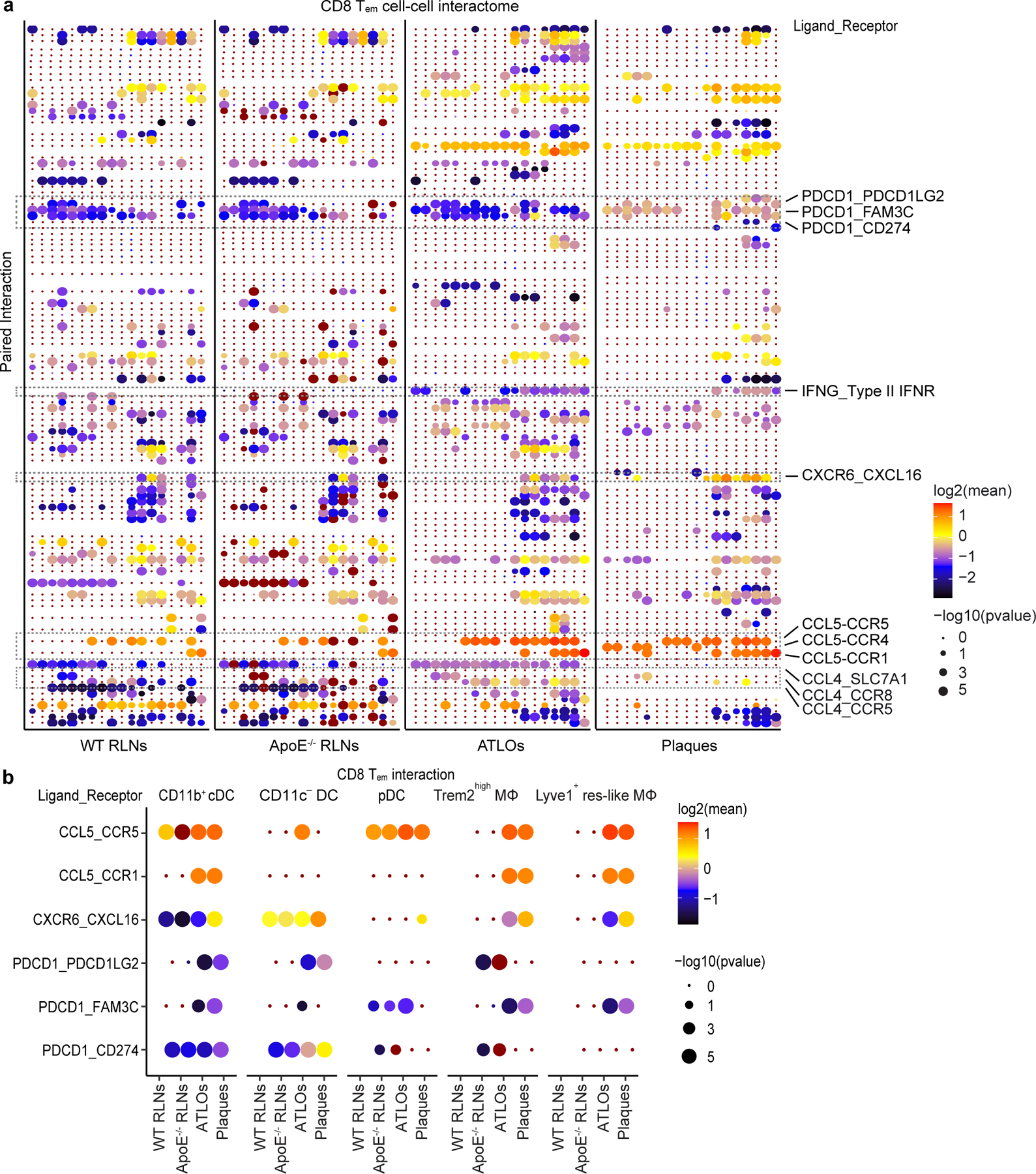
Predicted cell-cell interactome of CD8 T_em_ T cells with other immune cell subsets in WT RLNs, *Apoe*^*−/−*^ RLNs, ATLOs, plaques. **a**, The x axis represents the CD8 T_em_ T cell interaction with other immune cell subsets; the y axis depicts paired ligand-receptor interactions. Interactions contributing to T cell dysfunction in plaques when compared to WT RLNs are listed on the right. The size of circles is based on -log10 p values; color scheme is based on log2 value. **b**, Predicted interactions between CD8 T_em_ T cells with CD11b^+^ cDCs, pDCs, CD11c^−^ DCs, Trem2^hi^ macrophages, and Lyve1^+^ res-like macrophages in WT RLNs, *Apoe*^*−/−*^ RLNs, ATLOs, plaques. Color scale indicates CLR-normalized expression, dot size indicates significance (−log10 p value).

Our pairing approach together with scRNA-seq analyses of myeloid cells also allowed the identification of potential cell-to-cell interactions during atherosclerosis-specific T cell immune responses^21,35^ (Extended Data Fig. 9a). As CCL5 maintains CD8^+^ memory, we validated the algorithms by examining CCL5–CCR5 interaction in WT RLNs as positive controls: we observed high scores of CCL5–CCR5 interaction between CD8^+^ T_em_ cells and CD11b^+^ cDCs/plasmacytoid DCs (pDCs), but not CD11c^−^ DCs in WT RLNs, as expected (Extended Data Fig. 9a,b). CCL5–CCR5 interaction was high between CD8^+^ T_em_ cells and Trem2^hi^ macrophages/Lyve1^+^ tissue resident-like (res-like) macrophages in ATLOs and plaques (Extended Data Fig. 9b), indicating that the pro-inflammatory environments in both tissues participate in the maintenance of CD8^+^ T_em_ cell homeostasis. Interestingly, when compared to WT RLNs, plaque CD8^+^ T_em_ cells showed higher interaction of PDCD1 (also known as PD-1 or CD279) with their ligand/receptor, that is, PDCD1LG2 (PD-1 ligand 2, or CD273), FAM3C (family with sequence similarity 3 member c) and CD274 (PD-L1; Extended Data Fig. 9b) indicating notable exhaustion of plaque CD8^+^ T_em_ cells.

### Control of T cell checkpoints is broken in plaques

**Fig. 5 |:**
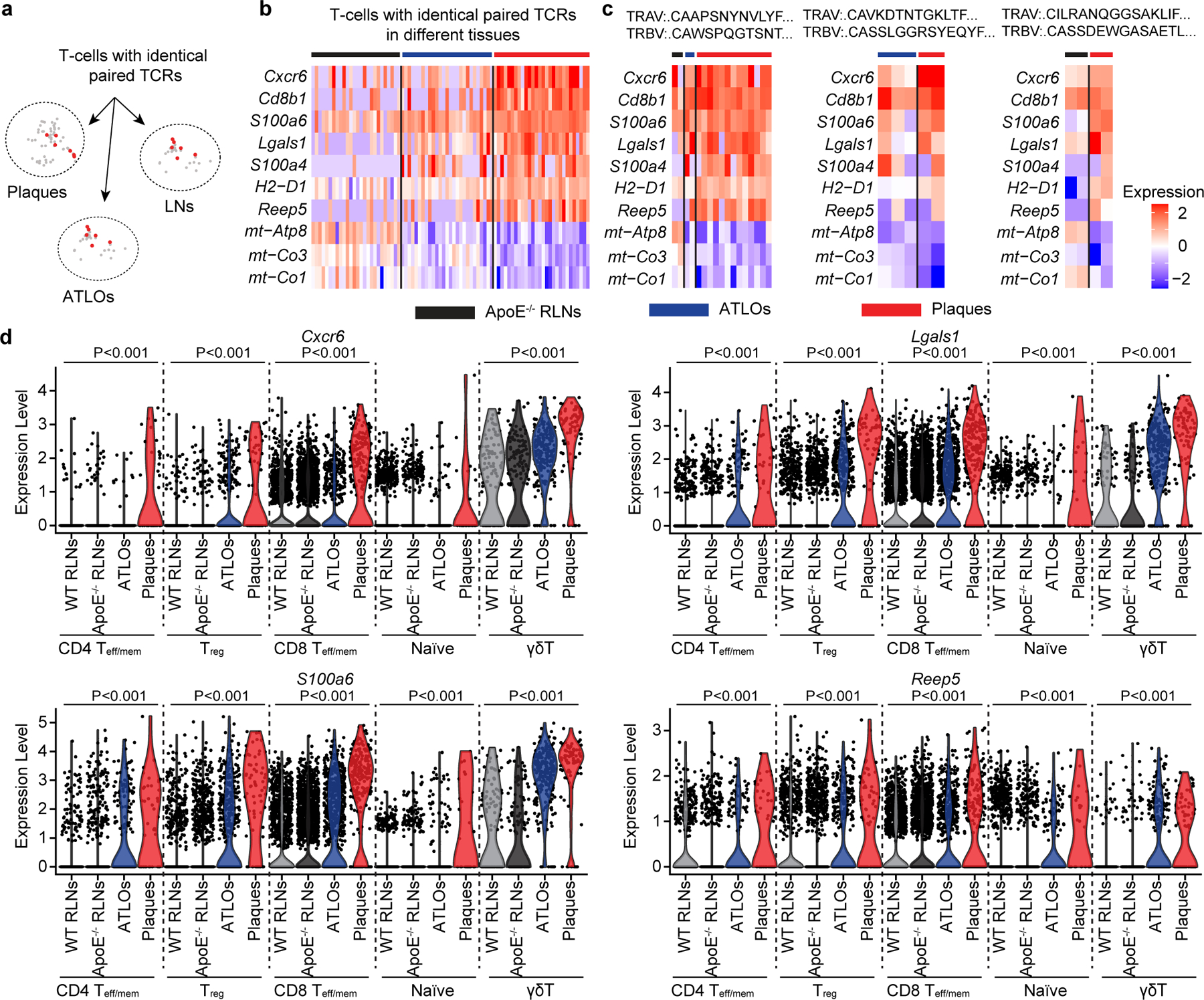
Delineation of plaque-inducible T cell transcript profiles. **a**, Schematic of experimental design. **b**, Heat maps of DEGs of pooled T cells carried identical TCRαβ pairs in different tissues. Each row represents one individual T cell. *n* = 28 T cells in plaques, 27 T cells in ATLOs and 26 T cells in *Apoe*^−/−^ RLNs. Two-sided Wilcoxon rank-sum test was used to identify DEGs and significant DEGs were defined by adjusted *P* value < 0.05. **c**, Heat maps of plaque-inducible genes in three individual T cell clones. T cells with identical TCR sequences from different tissues were compared. The CDR3 aa sequences of the paired TCRα/β pairs are listed on top of the heat maps. **d**, Violin plots showed *Cxcr6*, *Lgals1*, *S100a6* and *Reep5* gene expression by different T cell subsets. Each dot represents one cell. Kruskal–Wallis rank-sum test with Benjamini–Hochberg correction was used to analyze the differences among groups.

T cells adapt to different tissue microenvironments via reprogramming their gene expression signatures, which are critical for the maintenance of tissue-specific T cell tolerance^36,37^. To study mechanisms of tissue T cell homeostasis, we compared clonally expanded CD8^+^ T_eff/mem_ cells with identical TCRαβ chains in plaques, ATLOs and *Apoe*^−/−^ RLNs (Fig. 5a). We examined 81 CD8^+^ T_eff/mem_ T cells that shared identical paired TCRαβ sequences in different tissue environments in aged *Apoe*^−/−^ mice (Fig. 5a). Plaque-infiltrating T cells showed ten DEGs when compared to T cell clones located in ATLOs and RLNs, including seven upregulated genes (*Cxcr6*, *Cd8b1*, *S100a6*, *Lgals1*, *S100a4*, *H2-D1*, *Reep5*) and three downregulated genes (*mt-Atp8*, *mt-Co3*, *mt-Co1*; Fig. 5b). We termed these ten genes as the specific plaque-inducible gene signature (Fig. 5b). By further comparing three individual T cell clones, the plaque-inducible gene signature was observed in all three T cell clones (Fig. 5c), indicating that plaque-inducible gene signatures are independent of specific TCRαβ sequences. Of note, *Cxcr6* is reported to contribute to T cell activation during inflammation; *Lgals1* is involved in T cell apoptosis and is a T cell checkpoint inhibitor gene; *S100a6* contributes to T cell activation; *Reep5* is involved in regulating endoplasmic reticulum organization, but its role in T cell biology remains to be described; and *mt-Atp8, mt-Co3 and mt-Co1* genes are mitochondrial-related transcripts involved in energy metabolism. These data indicate that plaques impact T cell immunity via the regulation of transcripts related to activation, tolerance checkpoint and energy metabolism.

Plaque-inducible gene signatures may be imprinted on all plaque T cells and may not be limited to the 81 clonally expanded CD8^+^ T_eff/mem_ cells reported above. To clarify this possibility, we examined expression of *Cxcr6*, *Lgals1*, *Reep5* and *S100a6* genes in all T cell subsets (Fig. 5d). Different T cell subsets expressed different levels of *Cxcr6*, *Lgals1*, *S100a6* and *Reep5* in WT RLNs (Fig. 5d). These data revealed that these genes are differentially expressed by different T cell subsets under physiological conditions. Plaque T cells, including naїve T cells, CD4^+^ T_reg_ cells, CD8^+^ T_eff/mem_ cells, CD4^+^ T_eff/mem_ cells and γδ T cells expressed higher levels of *Cxcr6*, *Lgals1*, *Reep5* and *S100a6* versus T cell subsets in WT RLNs, *Apoe*^−/−^ RLNs and ATLOs (Fig. 5d). These data indicated that these genes are plaque-selective gene signatures which impact all plaque T cells.

### CD8^+^ tolerance is broken in human plaques

**Extended Data Fig. 10 |:**
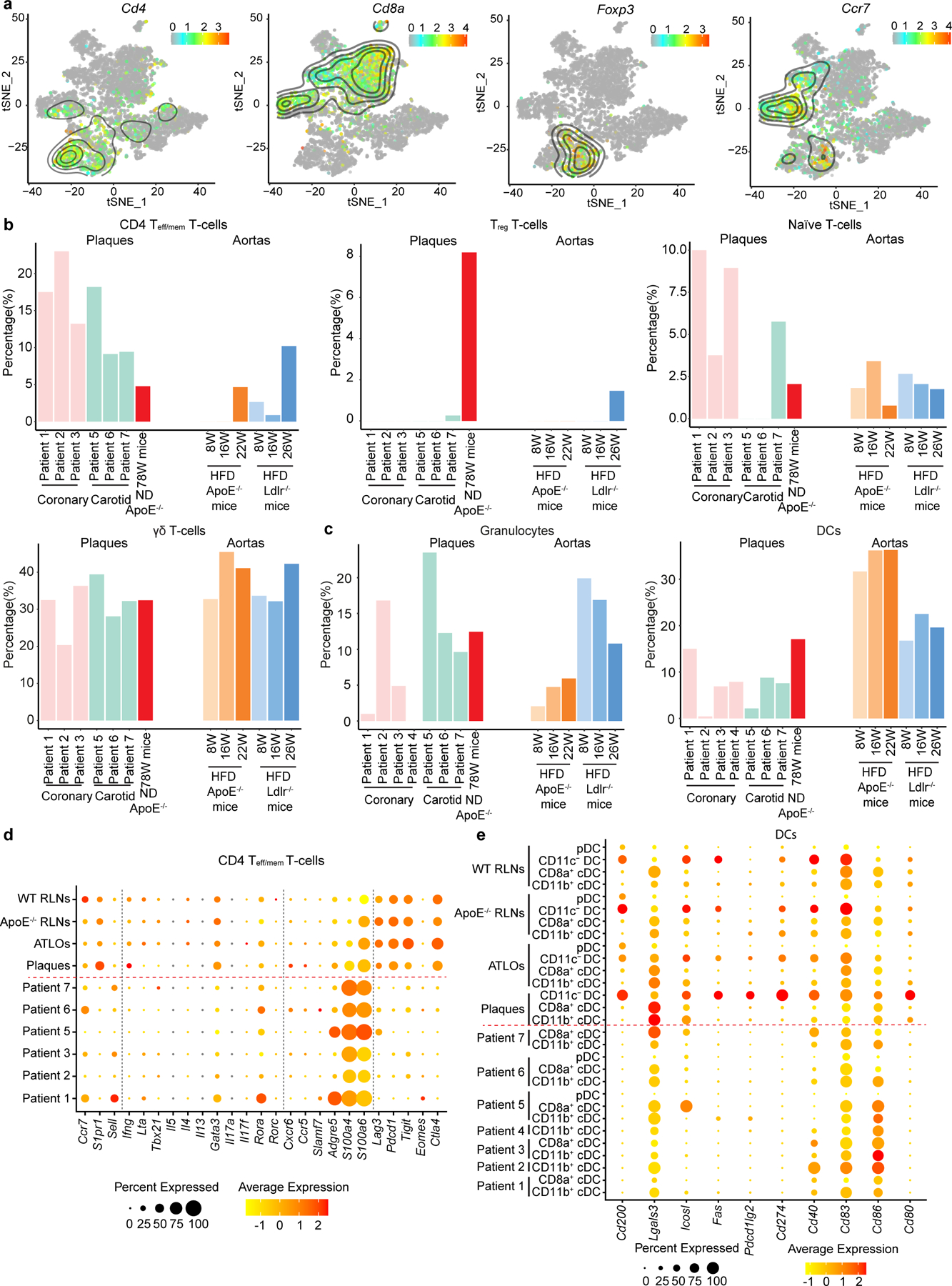
Comparison of human plaque T-/myeloid-cells and mouse plaque T-/myeloid-cells. **a**, tSNE plot shows the expression of T cell canonical marker genes *Cd4*, *Cd8a*, *Foxp3*, and *Ccr7* in the human-mouse integrated dataset as colored contour lines. Gene abbreviations on top of each panel; degree of relative expression indicated in colors with red designating high and gray designating low expression; **b**, Percentages of CD4 T_eff/mem_ T cells, T_reg_ T cells, naїve T cells, and γδ T cells in human plaques *vs* mouse plaques. **c**, Percentages of DCs and granulocytes in human plaques and mouse plaques. **d,e**, Dotplots display the tolerance-related genes in CD4 T_eff/mem_ T cells and DCs in human plaques and mouse plaques. Patient 1–4: human coronary plaques; patient 5–7: human carotid plaques (**b-e**).

**Fig. 6 |:**
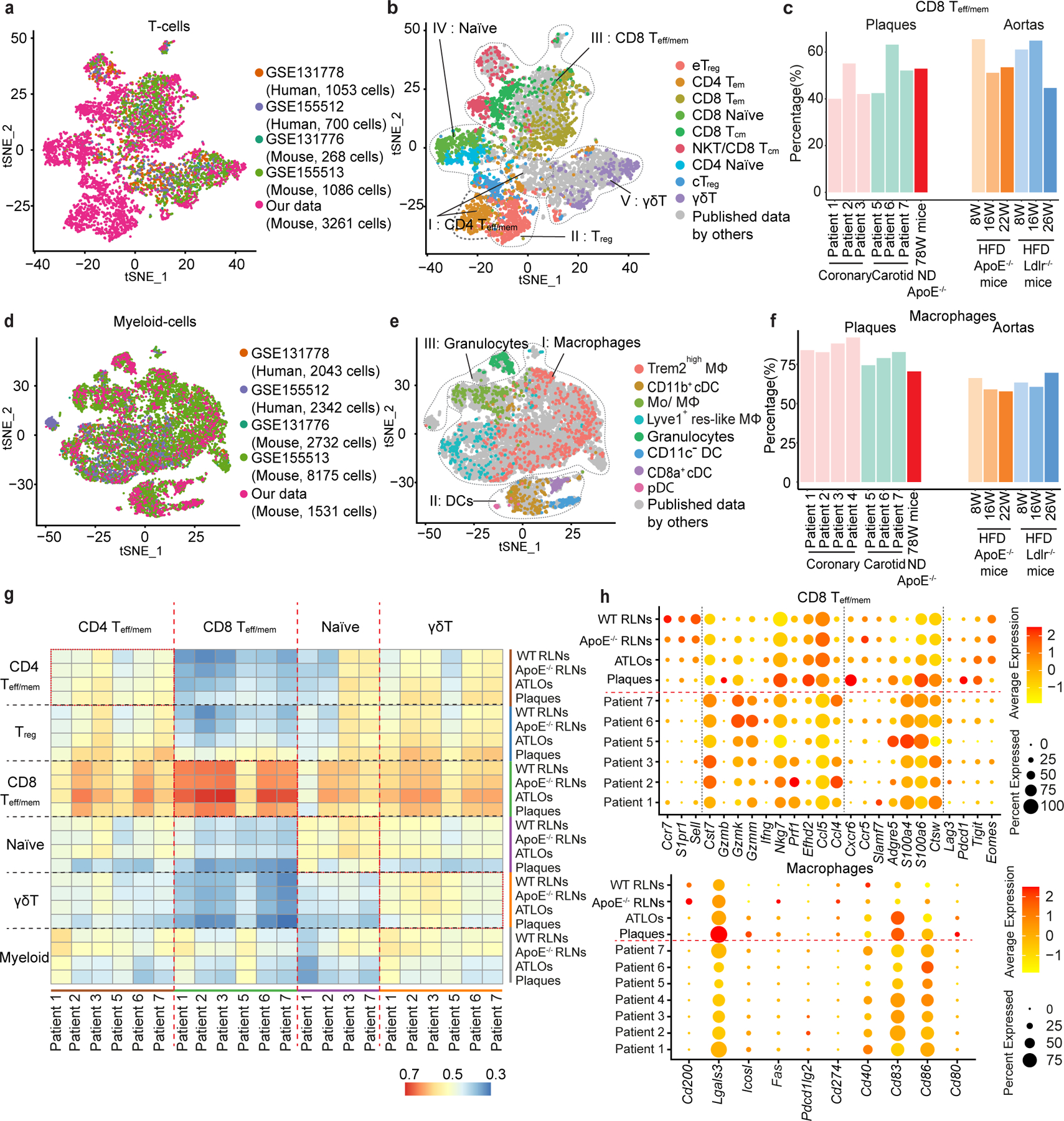
Comparing human plaque T cell and myeloid cell subsets with mouse plaque T cells. **a**, Integration analysis of human plaque T cells and mouse plaque/aorta T cells. *t*-SNE plot shows T cells obtained from publicly available databanks: GSE131778 and GSE155512: human coronary and carotid plaques; GSE131776: aorta of *Apoe*^−/−^ mice fed with a high-fat diet; GSE155513: the aorta of *Apoe*^−/−^ and *Ldlr*^−/−^ mice fed with high-fat diet; our data, aged *Apoe*^−/−^ mice fed with chow diet. **b**, Comparison of all T cell subsets in aged WT and *Apoe*^−/−^ mice and data from other public datasets. Cells from our data were colored; cells from other public databanks were labeled gray. T cell subsets were grouped to five T cell groups according to their functions in the *t*-SNE plot. Group I: CD4^+^ T_eff/mem_ cells; group II: T_reg_ cells; group III: CD8^+^ T_eff/mem_ cells; group IV: naïve T cells; group V: γδ T cells. **c**, Percentages of CD8^+^ T_eff/mem_ cells in human plaques, plaques of aged *Apoe*^−/−^ mice, in aortas of high-fat-diet-fed *Apoe*^−/−^ or *Ldlr*^−/−^ mice at different ages. **d**, Myeloid cells obtained from the same databanks were integrated and analyzed. **e**, Comparison of all myeloid cell subsets in aged WT and *Apoe*^−/−^ mice and data from other public datasets. Cells from our data were colored. Myeloid cell subsets were assembled into three major groups according to their functions in *t*-SNE analyses. Group I: macrophages, group II: DCs; group III: granulocytes. **f**, Percentages of macrophages in human plaques, in plaques of aged *Apoe*^−/−^ mice, in aortas of high-fat-diet-fed *Apoe*^−/−^ or *Ldlr*^−/−^ mice at different ages. **g**, Heat maps show the similarities between human plaque T cell subsets and mouse plaque T cell subsets. The top 50 subset-specific genes were used to calculate the Spearman correlation coefficient between human and mouse T cell subsets. Mouse T cell subsets from four tissues were compared with human T cell subsets of six individuals. **h**, Dot plot displays tolerance-related genes in CD8^+^ T_eff/mem_ cells and macrophages between human and mouse. Participants 1–4: human coronary plaques; participants 5–7: human carotid plaques (**c** and **f**–**h**).

We next compared our mouse data with published scRNA-seq datasets in human and mouse atherosclerosis by performing integration analyses, including human coronary plaques (GSE131778)^38^, human carotid plaques (GSE155512)^39^, and young or adult *Apoe*^−/−^ or *Ldlr*^−/−^ fed with a high-fat diet (GSE131776 and GSE155513)^38,39^. The shared T cell subsets and myeloid cell subsets in these arteries were identified across five datasets (Fig. 6a,d and Extended Data Fig. 10a). The T cell subsets and myeloid cell subsets that we observed in aged WT and *Apoe*^−/−^ mice matched the T cells and myeloid- cells in human plaques and in aortas of *Apoe*^−/−^ or *Ldlr*^−/−^ mice fed with a high-fat diet (Fig. 6b,e). To avoid confounding problems by looking at a few selected genes, we looked at T cells using expression of all 1,000–2,000 genes in each cell. We prepared integrated maps to examine both the similarities and dissimilarities of plaque T cell and myeloid cell compositions across species, diets, ages and mouse models^24,40^. First, we grouped all T cell subsets into five parent T cell subtypes as described above in Fig. 2b and all myeloid cells into three parent myeloid cell subtypes according to their biological functions, that is, macrophages, DCs and granulocytes (Fig. 6b,e). We observed that the percentages of antigen-experienced CD8^+^ T_eff/mem_ cells, but not other T cell subsets in plaques, were similar across species, diets and ages (Fig. 6c and Extended Data Fig. 10b) lending support to the dependability and robustness of our mouse–human comparisons regarding CD8^+^ T cells. Moreover, major similarities of plaque macrophage percentages were also observed between human and mouse plaques (Fig. 6f). However, DC and granulocyte percentages varied among different human samples, mouse models and ages (Extended Data Fig. 10c) indicating that comparisons need to be reexamined when stratification of a larger number of human atherosclerotic tissues at the scRNA-seq level will become possible in future studies to obtain more robust statistical analyses. To compare human plaque T cells versus mouse plaque T cell transcript profiles, we determined Spearman correlation coefficients between human and mouse T cell subsets based on their top 50 highly expressed mRNAs (Fig. 6g). Human plaque CD8^+^ T_eff/mem_ cells showed the highest correlation coefficient with their mouse CD8^+^ T cell counterparts (Fig. 6g), and the lowest correlation coefficient with mouse γδ T cells (Fig. 6g). These data indicate that human and mouse CD8^+^ T_eff/mem_ cells show major similarities at the levels of their mRNA expression signatures. We next examined expression of transcripts involved in tolerance regulation of T cells and APCs. Human plaque CD8^+^ T_eff/mem_ cells expressed similar levels of T cell egress-related genes (*Ccr7*, *S1pr1*, *Sell*) and activation-related transcripts (*Gzmk*, *Nkg7*, *Ccl5*, *S100a4*, *S100a6*, *Ctsw*) versus mouse plaque CD8^+^ T_eff/mem_ cells but these similarities were not observed in the corresponding CD4^+^ T cells (Fig. 6h and Extended Data Fig. 10d). These data support the notion that several mechanisms of tolerance breakdown in mouse plaque CD8^+^ T cells may also be applied to human plaque CD8^+^ T cells. Moreover, the expression of tolerance regulation-related genes in macrophages showed major similarities between human plaques and mouse plaques (Fig. 6h and Extended Data Fig. 10e), indicating antigen presentation to T cells is also compromised in human advanced plaques. These data indicate that CD8^+^ T cells and macrophages show major similarities in cell numbers and transcript profiles in human and mouse plaques (Fig. 6).

## Discussion

**Fig. 7 |:**
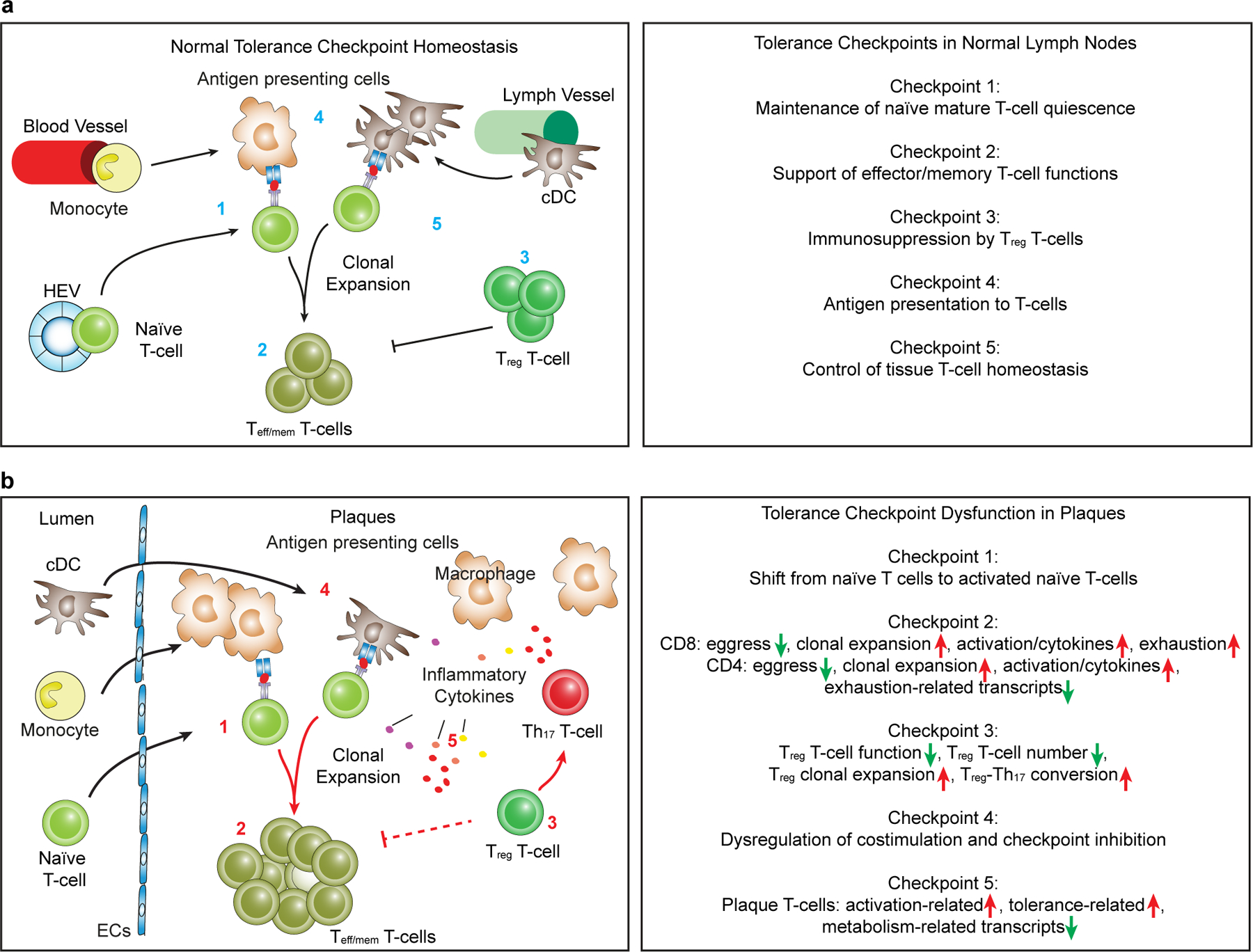
Landscape of T cell and myeloid cell tolerance breakdown in atherosclerosis. The immune system harbors a comprehensive system of multilayered checkpoints (see 1–5 in blue) to maintain tolerance in WT RLNs under physiological conditions to avoid autoimmune injury of self: (1) Maintenance of quiescence of naïve T cells; (2) support of effector/memory T cell functions; (3) immunosuppression by T_reg_ cells; (4) antigen presentation to T cells; (5) control of tissue T cell homeostasis. Checkpoints may be compromised at multiple levels (see 1–5 in red) in a tissue-specific manner in mouse advanced atherosclerosis as follows: *Apoe*^−/−^ RLNs may be compromised at checkpoint 2; ATLOs may be compromised at checkpoints 1, 2, 3 and 4; plaques may be compromised at all five checkpoints.

T cells participate in unresolvable inflammatory diseases as varied as cancers, infectious diseases, chronic degenerative brain diseases, autoimmune diseases and cardiovascular diseases^2,5,6,13,14^. T cell immunity is stringently controlled at multiple tolerance checkpoints to prevent autoimmune injury of self^12^. Our data support the possibility that peripheral T cell tolerance in atherosclerosis may be disrupted at multiple key checkpoints in tissue-specific and T cell-subtype-specific ways. Tolerance dysfunction in atherosclerosis may extend to checkpoints as varied as a shift from bona fide quiescence to an activated/memory-like phenotype within the naїve mature T cell pool in the circulation; clonal expansion of CD8^+^ T cells, CD4^+^ T cells and T_reg_ cells with plaques being the most prominent tissue affected followed by ATLOs and RLNs; major alterations of transcript profiles including genes regulating migration/egress/residency, T cell activation, maintenance of effector and memory functions and exhaustion; and T_reg_–Th_17_ cell conversion. Although human scRNA-seq data are not yet available for ATLOs and lymph nodes of patients burdened with atherosclerosis, data on coronary and carotid artery plaque CD8^+^ T cells suggest that plaque T cell tolerance dysfunction may be similar in mice and humans. Our data give comprehensive insight into potential mechanisms of peripheral tolerance breakdown in late-stage atherosclerosis and thus provide a robust blueprint to characterize atherosclerosis as an autoimmune T cell-driven disease that is associated with tolerance dysfunction (Fig. 7). As the current data expand insight into an underappreciated aspect of the pathogenesis of atherosclerosis, multiple targets of future therapeutic intervention including restoration of tolerance checkpoints and T cell therapeutics may emerge. However, we are aware of a caveat of the current data: Tolerance checkpoints work in tandem with multiple other immune responses and may be distinct for each checkpoint in tissue-specific and T cell-subtype-specific ways. As our data are based on transcript profiles, FACS identification of T cell subsets and immunohistochemical analyses reported previously^7,8,18,19^ and expanded here (Extended Data Fig. 2c and Fig. 4c,d), the challenges for future work will be to intervene experimentally in mice and human clinical studies to determine which tolerance checkpoints affect atherosclerosis progression similar to studies that have been conducted in various forms of human cancers^41^. Moreover, new immune tolerance checkpoints not described here may be discovered in the future.

That three major T cell subsets undergo clonal expansion in late-stage atherosclerosis deserves special attention. This observation indicates that atherosclerosis/hyperlipidemia-related autoantigens may give rise to proatherogenic autoreactive CD8^+^ T cells and that atherosclerosis autoimmune responses involve the generation of autoreactive T_reg_ cells and CD4^+^ T cells. These data lead us to propose that next-generation antigen-specific T_reg_ cells including chimeric antigen-receptor T cells to treat atherosclerosis should be considered in future experimental studies^42,43^. Many T cells recognizing self-antigens are T_reg_ cells^10^. However, in the context of atherosclerosis, T_reg_ cells become unstable and may switch sides from immunosuppressive to immunostimulatory T cells during T_reg_/TH_17_ cell conversion: (1) they acquire additional transcription factors in addition to the hallmark transcription factor FoxP3 (plasticity); and (2) they lose FoxP3 and CD25, the high-affinity IL-2 receptor (T_reg_ cell instability) generating cells termed exT_reg_ cells^32,44^. Although we did not conduct rigorous lineage-tracking experiments, our present data suggest that exT_reg_ cells acquire a TH_17_-like cell transcriptome. Using tetramers, Kimura et al.^45^ showed that most APOB epitope-specific CD4^+^ T cells in blood expressed FoxP3 in women without cardiovascular disease but coexpressed FoxP3 and RORγt in women with cardiovascular disease. Our current data in mice support the notion that T_reg_ cells can switch to ROR-γt^+^ T cells in plaques and show that the breakdown of tolerance may be strictly tissue specific: most evident in plaques, then in ATLOs and to a lesser degree in RLNs and the circulation. Compromised tolerance checkpoints have been extensively studied in various clinically important diseases, including autoimmune diseases and cancers^46–48^. These studies identified powerful checkpoint inhibition therapies. However, systemic targeting of two checkpoint inhibitors shown to be highly effective in cancer (that is, anti-PD-1/PD-L1 and anti-CTLA4) accelerated atherosclerosis development in mice^49–52^ and ischemic heart disease in humans^53^. Here, we provide a potential mechanism for this effect: anti-PD-1 or anti-PD-L1 unleash antigen-experienced T cells to recognize atherosclerosis-specific epitopes and thus may exacerbate and accelerate atherosclerosis. However, many more new checkpoints are being discovered beyond the PD-1/PD-L1 pair and CTLA4, and these newly discovered checkpoints^54^ need to be reexamined for their effect on atherosclerosis in the future to fully understand the relationship between tolerance-directed therapies in cancer versus atherosclerosis.

Whether plaque-infiltrating T cells recognize atherosclerosis disease-relevant autoantigens is one of the central unresolved questions in atherosclerosis. Although our approach did not yet identify the relevant autoimmune epitopes, our data provide a tangible blueprint for the future to identify these epitopes as we have defined three diverse T cell subsets with distinct roles in the atherosclerosis immune response as being clonally expanded. Interestingly, two previous studies have shown APOB epitope-specific CD4^+^ T cells in atherosclerotic mice and human blood^32,45^, and these T cells expressed the TRBV31 family. Moreover, inhibition of TRBV31^+^ T cells reduced atherosclerosis^55^. Thus, our current unbiased approach calls for the search for bona fide T cell-dependent peptide antigens in mouse and human atherosclerosis.

Finally, our data have translational potential. A tolerogenic vaccine might specifically tolerize atherosclerosis autoantigen-specific T cells. In order to build such a vaccine, the epitopes must be identified. Prediction software like GLIPH2 (ref. ^56^) can predict potential epitopes based on TCRα and TCRβ sequences. Successful tolerance induction has already been achieved in autoimmune celiac disease^57^. Another potential approach could be to engineer T_reg_ cells^43^ using our TCRα and TCRβ chains. Such a cell-based immunotherapy^58^ would be expected to dampen the autoimmune response that exacerbates atherosclerosis.

## Methods

### Mice

*Apoe*^−/−^ mice on a C57BL/6 background and C57BL/6 WT mice were housed in the specific pathogen-free animal facilities of Munich University with a 12-h light/dark cycle, in an air-conditioned room (23 °C and 60% relative humidity). All mice were 78–85 weeks old and were maintained on a standard rodent chow. Animal procedures were approved by the Regierung of Oberbayern according to the guidelines of the local Animal Use and Care Committee and the National Animal Welfare Laws.

### Tissue collection and processing

Mice were euthanized by ketamine hydrochloride and xylazine hydrochloride. Blood was collected by cardiac puncture. Perfusion was performed from the left ventricle with 10 ml 5 mM EDTA buffer, 20 ml PBS and 20 ml FACS buffer. RLNs were collected under a dissecting microscope. To collect plaques and ATLOs, we followed the method described previously^7,20^. In short, adipose tissue and paraaortic lymph nodes were carefully removed. The whole aorta was dissected and collected in cell culture dishes with pre-cooled FACS buffer. The aorta was opened in the longitudinal direction, and the plaque tissues were carefully removed using curved forceps (Dumont 5/45, Fine Science Tools) under the dissecting microscope. The remaining aorta was collected as ATLOs. Three separated cohorts of mice were used: (1) pools of three *Apoe*^−/−^ and three WT mice were used to collect plaques, ATLOs and RLNs, and pools of five *Apoe*^−/−^ and five WT mice were used to collect blood in the first cohort; (2) cell hashtags (hashtags, BioLegend, TotalSeq C) were used to label four *Apoe*^−/−^ and four WT mice individually to collect ATLOs and RLNs; and (3) pools of five *Apoe*^−/−^ mice were used to collect plaque tissues.

### Single-cell isolation for single-cell profiling

Blood samples were centrifuged at 4 °C and 8 ml ammonium-chloride-potassium buffer was added to lyse red blood cells at room temperature. ATLOs and plaques were cut into small pieces and digested with enzyme cocktail (400 U ml^−1^ collagenase type I, 10 U ml^−1^ collagenase type XI, 60 U ml^−1^ hyaluronidase, 60 U ml^−1^ DNase I and 20 mM HEPES in DPBS) for 40 min at 37 °C with slow shaking. Cell suspensions were filtered through a 100-μm strainer and rinsed with pre-cooled FACS buffer. RLNs were cut into small pieces and mashed on the 100-μm strainer with the plunger of the syringe, then rinsed with pre-cooled FACS buffer. Following centrifugation and resuspension, cells of each tissue were stained with fixable viability dye (1:1,000 dilution; eBioscience) in PBS at 4 °C for 20 min. After washing and blocking steps, cells were stained with CD45 labeled in fluorescence (1:200 dilution; BD Bioscience, clone 30-F11, for pooling cohort) or CD45.2 (1:200 dilution; BioLegend, clone 104, for hashtag cohort) with or without hashtagged CD45/MHCI antibody (1:50 dilution; BioLegend, TotalSeq C) at 4 °C for 30 min. Single CD45^+^ live cells were sorted using a flow cytometer (FACSAria III, BD Biosciences).

### Library preparation for gene expression and T cell antigen receptor sequencing

Cell density and live-cell percentages of sorted CD45^+^ cells were measured by an automated cell counter (TC20, Bio-Rad). Cell concentrations were adjusted to 700–1,200 cells per microliter according to the protocol of 10X Genomics 5′ library & gel bead kit. Single-cell suspensions were mixed with nuclease-free water and 5′ single-cell RNA master mixture, then loaded into a Chromium chip with barcoded gel beads and partitioning oil. The chip was placed in the Chromium controller or Chromium X to generate gel beads in emulsion (GEMs). cDNA was obtained from 100–180 μl GEMs/sample by reverse-transcription reactions: 53 °C for 45 min, 85 °C for 5 min, then maintained at 4 °C. cDNA products were purified and cleaned using Dynabeads. cDNA was amplified by PCR: 98 °C for 45 s; 98 °C for 20 s, 67 °C for 30 s, 72 °C for 1 min and amplified for 13–16 cycles; then, 72 °C for 1 min. Amplified PCR products were cleaned and purified using SPRIselect reagent kit (B23317, Beckman Coulter). The concentration of cDNA library was determined by Qubit dsDNA HS Assay Kit (Invitrogen).

### Construction of gene expression next-generation sequencing libraries

A 50 ng mass of cDNA library per sample or 20 μl of hashtagged cDNA library per sample were used to construct 5′ gene expression libraries by using the Chromium single-cell 5′ library construction kit (1000020, 10X Genomics) or the Chromium next GEM single-cell 5′ HT kit (1000374, 10X Genomics). Briefly, following the fragmentation (32 °C for 5 min), end repairing and A-tailing (65 °C for 30 min then hold at 4 °C) steps, all expression DNAs were fragmented. Fragments 300–500 bp in length were selectively purified by using double-sided size selection of the SPRIselect kit (B23317, Beckman Coulter). Next-generation sequencing (NGS) adaptors were added to the PCR fragments following adaptor ligation PCR amplification using the Chromium i7 multiplex kit (120262, 10X Genomics) or dual index plate TT set A kit (3000431, 10X Genomics): 98 °C for 45 s; 98 °C for 20 s, 54 °C for 30 s, 72 °C for 20 s and 15 cycles or 13 cycles; and 72 °C for 1 min. The ready-to-load gene expression NGS libraries were cleaned and purified using the SPRIselect kit. The size and the concentration of gene expression NGS libraries were determined by the fragment analyzer (Advanced Analytical Technologies) and the NEBNext library quantification kit (E7630L, New England Biolabs), respectively.

### T cell antigen receptor next-generation sequencing library construction

A total of 2 μl of cDNA library of each sample was used for two rounds of TCR amplification using TCR-specific PCR primers by using the Chromium single-cell V(D)J enrichment kit (1000071, 10X Genomics). The first-round enrichment PCR was performed under the following conditions: 98 °C for 45 s; 98 °C for 20 s, 67 °C for 30 s, then 72 °C for 1 min, amplified for 10 cycles; then 72 °C for 1 min. The first-round PCR products were cleaned and purified by using the SPRIselect kit. The second-round enrichment PCR was performed as follows: 98 °C for 45 s; 98 °C for 20 s, 67 °C for 30 s, 72 °C for 1 min, repeat for 10 cycles, then, 72 °C for 1 min. After clean-up and purification using the SPRIselect kit, NGS adaptors were added to the PCR fragments by adaptor ligation using the Chromium i7 multiplex kit (120262, 10X Genomics) or the dual index plate TT set A kit (3000431, 10X Genomics): 98 °C for 45 s; 98 °C for 20 s, 54 °C for 30 s, 72 °C for 20 s, 8 cycles or 12 cycles; then 72 °C for 1 min. The ready-to-load TCR NGS libraries were cleaned and purified using the SPRIselect kit. The size and concentration of TCR libraries were determined by the fragment analyzer (Advanced Analytical Technologies) and the NEBNext library quantification kit (E7630L, New England Biolabs), respectively.

### Next-generation sequencing

For the first cohort of pooled samples, 5′ gene expression libraries and TCR libraries were sequenced separately at IMGM (https://www.imgm.com/). Around 1% PHiX control library spike-in (Illumina) was added to 5′ gene expression libraries and sequenced by a NextSeq high-output reagent kit v.2.5 (150 cycles) using the Nextseq 500 platform (Illumina). Sequencing was performed as an asymmetrical manner: read 1 was adjusted to 26 bp, read 2 to 98 bp and the index 1 to 8 bp. TCR libraries together with 1% PHiX control library spike-in were sequenced in a symmetrical manner (150 bp + 150 bp, paired end). For the second cohort of hashtagged samples, 5′ gene expression libraries, TCR libraries and cell hashtag libraries were sequenced together using the NovaSeq 6000 platform at IMGM (https://www.imgm.com/). Three types of libraries were pooled at the ratio of 40:10:1. Sequencing was performed in an asymmetrical manner: read 1 was adjusted to 28 bp plus 10 bp for i7 index, 10 bp for i5 index, and read 2 to 90 bp. For the third cohort of pooled plaques, the 5′ gene expression library was sequenced using the NovaSeq 6000 platform as described above.

### Single-cell sequencing data pre-processing analysis

The sequencing raw base call (BCL) files were demultiplexed with Cell Ranger (version 3.1.0 or version 7.0.1) using the Linux system (Intel(R) Xeon(R) E5–1650 v3 processor, Cores: 6, Threads: 12). For the 5′ gene expression sequencing data, reads were aligned to mouse mm10 reference transcriptome using STAR aligner in the ‘cellranger count’ or the ‘cellranger multi’ function. For the V(D)J libraries, reads were aligned to the GRCm38 –3.1.0 reference transcriptome using the ‘cellranger vdj’ or ‘cellranger multi’ function to perform sequence assembly and paired clonotype calling in Cell Ranger. The output raw barcodes, features and matrix files were loaded into R (version 4.2.1) and processed with the package Seurat (version 4.3.0)^22,23^. Cells with less than 200 genes and genes detected in less than three cells were removed as the first step of quality control. Low-quality (that is, high percentage of mitochondrial genes) and duplicated (that is, high number of gene per cell) cells were filtered out according to the following criteria: for blood samples, cells with >3,000 detected genes and >10% of mitochondrial genes were removed; for WT RLNs, *Apoe*^−/−^ RLNs and ATLOs, cells with >4,000 detected genes and >8% of mitochondrial genes were removed; for plaques, cells with >5,000 detected genes and >8% of mitochondrial genes were removed. Data were normalized and log transformed to remove the confounding of different sequence depths. After the quality-control steps, data were ready for further analysis.

### Gene expression landscapes for T cells and myeloid cells

The top 2,000 highly variable genes across cells were used to perform canonical correlation analysis to identify anchor cells for data integration in an unsupervised strategy. Then, integrated data were scaled to get rid of the effect of highly expressed genes for downstream analysis. For cell clustering, in blood samples, the top 20 significant principal components (PCs) were used to construct the shared nearest-neighbor (SNN) graph and the top 20 significant PCs were used for *t*-SNE visualization. In RLNs, ATLOs and plaques, the top 10 significant PCs were used to construct the SNN graphs, and the top 20 significant PCs were used for *t*-SNE clustering. Cell-type-specific markers were used to define immune cell subsets, that is, *Cd3e* for T cells, *Cd19* and *Cd79a* for B cells, and *Cd68 and Itgam* for myeloid cells. To analyze T cells and myeloid cells in a higher resolution, T cells and myeloid cells were extracted separately from total CD45 cells for further analysis. The top 15 PCs were used to construct the SNN graphs, and the top 20 PCs were used to generate *t*-SNE plots for T cell-subset clustering. The top 20 PCs were used to construct the SNN graphs, and the top 20 PCs were used for *t*-SNE plots for myeloid cell-subset clustering. DEGs were defined by fold change > 0.25 and adjusted *P* < 0.05.

### T cell antigen receptor repertoire analysis

T cell receptor beta-chain variable (TRBV) family percentage was calculated as follows: the individual TRBV family count / the total TRBV families count × 100%. The frequencies of TRBV-J family combination usage and the overlapped CDR3 sequences among tissues were summarized and plotted with the Circlize package (version 0.4.13). The Shannon entropy and Gini index were calculated by philenthropy (version 0.4.0, function: H(x)) and the edgeR (version 3.26.8, function: gini(x)) package, respectively. All samples were randomly down-sampled to the same sequence depth and downsampling was repeated 1,000 times for the Shannon entropy and Gini index calculations.

### Pairing the T cell antigen receptor with gene expression landscape analysis

Single-cell TCR data were extracted from the outputs after running single-cell 5′ V(D)J library sequencing data using the Cell Ranger ‘cellranger vdj’ or ‘cellranger multi’ function. The reconstructed TCR contigs were filtered by limiting to productive TCRα and TCRβ chains. The unique cell barcode sequences were used to pair two libraries, and TCRαβ sequences and 5′ gene expression sequencing metadata were integrated by Seurat. The integrated dataset was used to calculate the percentage of expanded and non-expanded T cells in *t*-SNE plots. More than two cells with identical V family and TCRαβ CDR3 sequences, but with different cell barcode sequences were considered as clonally expanded T cells. The pairing of gene expression and TCR repertoire datasets were manually double checked for the typical T cell markers, including *Cd4*, *Cd8*, *Cd44*, *Sell*, *Ccr7* and *Gzmk* to estimate the accuracy of pairing algorithm. By checking all 547 clonally expanded T cells, 10 of 547 T cells were corrected for cell-subtype definition (see all ten cells listed in Table 1).

### Analysis of tolerance-related transcript expression

Heat maps, Violin plots and *t*-SNE plots were used to show the expression of tolerance-related transcripts in selected cell subsets. Heat maps, violin plots and *t*-SNE plots were generated by the built-in functions of Seurat, the ggpubr package (0.4.0) and the ggplot2 package (3.3.5). Cell–cell interaction was performed with CellPhoneDB (version 4)^21^.

### Integration analysis of human and mouse scRNA-seq data

Human atherosclerotic plaque scRNA-seq data and mouse aorta scRNA-seq data were downloaded from publicly available datasets, including human carotid plaques (GSE155512), human coronary plaques (GSE131778) and the total aortas of 8- to 26-week-old *Ldlr*^−/−^ mice fed with a high-fat diet or *Apoe*^−/−^ mice fed with a high-fat diet (GSE155512, GSE155513). Low-quality cells were removed according to their own filtering criteria^38,39^. Human genes were converted to mouse genes by using the biomaRt package (version 2.40.5). Genes that could not be converted were excluded from further analysis. The top 20 PCs were used to construct SNN graphs, and the top 20 PCs were used to separate T cells, macrophages/myeloid cells and B cells in *t*-SNE plots. T cells and myeloid cells were extracted from each database, and then integrated with our current datasets. Human and mouse T cell or myeloid cell subsets in *t*-SNE plots were generated as described above.

### Immunofluorescence staining

Mouse aortas were prepared and embedded in Tissue-Tek (Sakura Finetek), and serial 10-μm-thick frozen tissue sections were prepared. Immunostainings were performed using following marker antibodies: anti-mouse CD3e (145–2C11, BD; 1:100 dilution), anti-mouse Foxp3 (ab75763, Abcam; 1:100 dilution) and DAPI for DNA. Secondary antibodies were used as previously described^7^. For negative controls, stainings were performed without primary antibodies or isotype controls. Stained sections were analyzed using a Leica SP8 ×3 microscope (Leica microsystems, Germany). Images were acquired with identical microscope settings using sequential channel acquisitions to avoid cross-talk between fluorophores. All images were prepared as TIF files by Fiji (ImageJ, National Institutes of Health) or Leica LAS-X (V3.5) software and exported into Adobe Illustrator CS6 for figure arrangements.

### Flow cytometry analyses

Cells for flow cytometry were prepared as described^7^: cells were stained with fixable viability dye for 20 min at 4 °C, then treated with purified anti-mouse CD16/CD32 monoclonal antibody to block Fc receptors. Next, cells were incubated with antibodies for 25 min at 4 °C, then fixed the cells 40 min at 4 °C, followed by antibody incubation for 45 min at 4 °C in fixation and permeabilization buffer (eBioscience) for intracellular staining. After incubation, the samples were washed and resuspended in FACS buffer before analysis. Antibodies used to define T cell subsets were used as described^7^. The following reagents/antibodies were used for flow cytometry: Fixable Viability Dye (Fluor 780, Bioscience, 65–0865–14; 1:1,000 dilution); CD16/CD32 (unconjugated, eBioscience, 14–0161–82, clone 9E9; 1:250 dilution); CD45 (PerCP-Cy5.5, Bioscience, 45–0451–82, clone 30-F11; 1:200 dilution); TCRβ (BV605, BioLegend, 109241, clone H57–597; 1:200 dilution); CD4 (BV421, BioLegend, 100438, clone GK1.5; 1:200 dilution); CD8a (Alexa Fluor 700, BioLegend, 100730, clone 53–6.7; 1:200 dilution); Foxp3: (PE, Bioscience, 12–5773–82, clone FJK-16s; 1:200 dilution); CD44 (APC, BioLegend, 103012, clone IM7; 1:200 dilution); CD62L (FITC, Bioscience, 11–0621–82, clone MEL-14; 1:200 dilution). For each experiment, compensation was developed using single staining controls and compensation beads (Invitrogen, 01–2222–41). Data were expressed as the percentages of total T cells. Data were acquired using a BD LSRFortessa (BD Bioscience), and data were analyzed using FlowJo (v.10.8, BD)

### Statistical analysis

Data were analyzed using the packages ‘stats’ and ‘fifer’ in R (version 4.2.1; R Foundation for Statistical Computing), GraphPad Prism (version 9.4.1) and SPSS v.29 (IBM). Descriptive statistics for discrete variables included mean and standard error of mean, while categorical variables are reported as proportion of the total, unless differently indicated. For comparisons of discrete variables, data distribution was tested by Shapiro–Wilk test. For data that followed a Gaussian distribution, a one-way analysis of variance with Bonferroni post hoc test was used to perform statistical analysis among multiple groups. As most datasets did not follow Gaussian distribution, the difference between two groups was compared by two-sided Wilcoxon rank-sum test and difference between three or more groups was analyzed by non-parametric Kruskal–Wallis *H* test with Dunn’s post hoc test for pairwise comparisons. For categorical variables, comparisons were formally analyzed by Pearson’s Chi-square test and followed by post hoc Fisher’s exact test with the false discovery rate approach suggested by Benjamini–Hochberg to reduce the inflation of the alpha error. Differences were considered significant at a two-tailed *P* value < 0.05.

### Reporting summary

Further information on research design is available in the Nature Portfolio Reporting Summary linked to this article.

## Extended Data

## Supplementary Material

Source Data Extended Data Fig.2

Source Data Extended Data Fig.3

Source Data Extended Data Fig.4

Source Data Fig.1

Source Data Extended Data Fig.10

Source Data Fig.4

Source Data Fig.6

Source Data Fig.2

Supplementary Table 3

Supplementary Fig.1

Supplementary Table 1

Supplementary Table 2

## Figures and Tables

**Table 1:** Correction of subtype definition of 10 of 547 clonally expanded T cells

No.	Cell subsets determined by algorithm	Marker gene expression						Corrected cell subsets	Note
		Cd4	Cd8a	Cd44	Sell	Ccr7	Gzmk		
1	CD8^+^ naïve	0	4	5	0	0	2	CD8^+^ T_em_	*Cd8* ^+^ *Cd44* ^+^ *Sell* ^−^ *Ccr7* ^−^ *Gzmk* ^+^
2	CD8^+^ T_em_	0	1	3	3	1	4	CD8^+^ T_cm_	*Cd8* ^+^ *Cd44* ^+^ *Sell* ^+^ *Ccr7* ^+^ *Gzmk* ^+^
3	CD8^+^ T_em_	0	3	0	2	2	3	CD8^+^ T_cm_	*Cd8* ^+^ *Sell* ^+^ *Ccr7* ^+^ *Gzmk* ^+^
4	CD8^+^ T_em_	0	3	2	1	1	6	CD8^+^ T_cm_	*Cd8* ^+^ *Cd44* ^+^ *Sell* ^+^ *Ccr7* ^+^ *Gzmk* ^+^
5	CD8^+^ T_em_	3	0	1	0	0	0	CD4^+^ T_em_	*Cd4* ^+^ *Cd8* ^−^ *Cd44* ^+^ *Sell* ^−^ *Ccr7* ^−^ *Gzmk* ^−^
6	CD8^+^ T_em_	1	0	1	0	0	0	CD4^+^ T_em_	*Cd4*^+^*Cd8*^−^Cd44^+^*Sell*^−^*Ccr7*^−^*Gzmk*^−^
7	CD8^+^ T_cm_	2	0	0	0	0	0	CD4^+^ T_cm_	*Cd4* ^+^ *Cd8* ^−^ *Sell* ^−^ *Ccr7* ^−^ *Gzmk* ^−^
8	CD4^+^ naïve	3	0	4	0	0	0	CD4^+^ T_em_	*Cd4* ^+^ *Cd44* ^+^ *Sell* ^−^ *Ccr7* ^−^
9	CD4^+^ naïve	0	0	2	0	0	0	CD4^+^ T_em_	*Cd44* ^+^ *Sell* ^−^ *Ccr7* ^−^
10	CD4^+^ naïve	1	0	1	0	0	0	CD4^+^ T_em_	*Cd44* ^+^ *Sell* ^−^ *Ccr7* ^−^

10 of 547 (1.828%) were classified as wrong T cell subsets. We estimated that 98.172% accuracy of the pairing of gene expression and TCR repertoire algorithm was achieved.

## Data Availability

The count matrix and metadata of scRNA-seq data and contig annotations for scTCR-seq are deposited in Figshare (https://doi.org/10.6084/m9.figshare.21900735.v2). The paired CDR3 sequencing data are available from the corresponding authors on reasonable request. The public mouse and human atherosclerosis data used in our study can be downloaded from the Gene Expression Omnibus under accession numbers GSE155512, GSE131778, GSE155512, GSE155513. Source data are provided with this paper.
