# Supplementary material for: Pairing of single-cell RNA analysis and T cell antigen receptor profiling indicates breakdown of T cell tolerance checkpoints in atherosclerosis
Source: Nat Cardiovasc Res. Author manuscript; Available in PMC 2023 Aug 24. (PMC10448629; doi:10.1038/s44161-023-00218-w)
Supplement: Supplementary Table 3 [file NIHMS1913298-supplement-Supplementary_Table_3.pdf]

Supplementary Table 3. DEGs of different T-cell types between WT blood and ApoE$^{-/-}$ blood. Data were calculated by the FindMarkers function in Seurat , two-sided Wilcoxon-Rank Sum test was used to identify differentially expressed genes (DEGs). Genes with avg_log2FC>0,25 and adjusted P value <0.05 were considered as significant DEGs. Bonferroni correction was performed to adjust P values. avg_log2FC: log fold-chage of the average expression between the two groups; pct.1: the percentage of cells where the feature is detected in the first group; pct.2: the percentage of cells where the feature is detected in the second group; p_val_adj: adjusted p value.

Celltype	Gene symbol	p_val	avg_log2FC	pct.1	pct.2	p_val_adj
CD8 Naïve	Slc1a5	3,39E-12	-0,7555963	0,027	0,311	4,41E-08
CD8 $T_{eff/mem}$	Pglyrp1	6,86E-08	-0,6776942	0,325	0,603	0,0008922
CD8 $T_{eff/mem}$	Hbb-bs	1,40E-07	-0,5645819	0,06	0,258	0,0018272
CD8 $T_{eff/mem}$	Gnai2	1,98E-07	0,4894749	0,795	0,67	0,0025814
CD8 $T_{eff/mem}$	Zmat2	3,98E-07	-0,5293611	0,19	0,416	0,0051797
CD8 $T_{eff/mem}$	Gpr183	1,47E-06	-0,5387118	0,355	0,627	0,0191293
CD8 $T_{eff/mem}$	Slc1a5	2,51E-06	-0,4975133	0,09	0,278	0,0326721
