# Supplementary material for: Pairing of single-cell RNA analysis and T cell antigen receptor profiling indicates breakdown of T cell tolerance checkpoints in atherosclerosis
Source: Nat Cardiovasc Res. Author manuscript; Available in PMC 2023 Aug 24. (PMC10448629; doi:10.1038/s44161-023-00218-w)
Supplement: Supplementary Fig.1 [file NIHMS1913298-supplement-Supplementary_Fig_1.pdf]

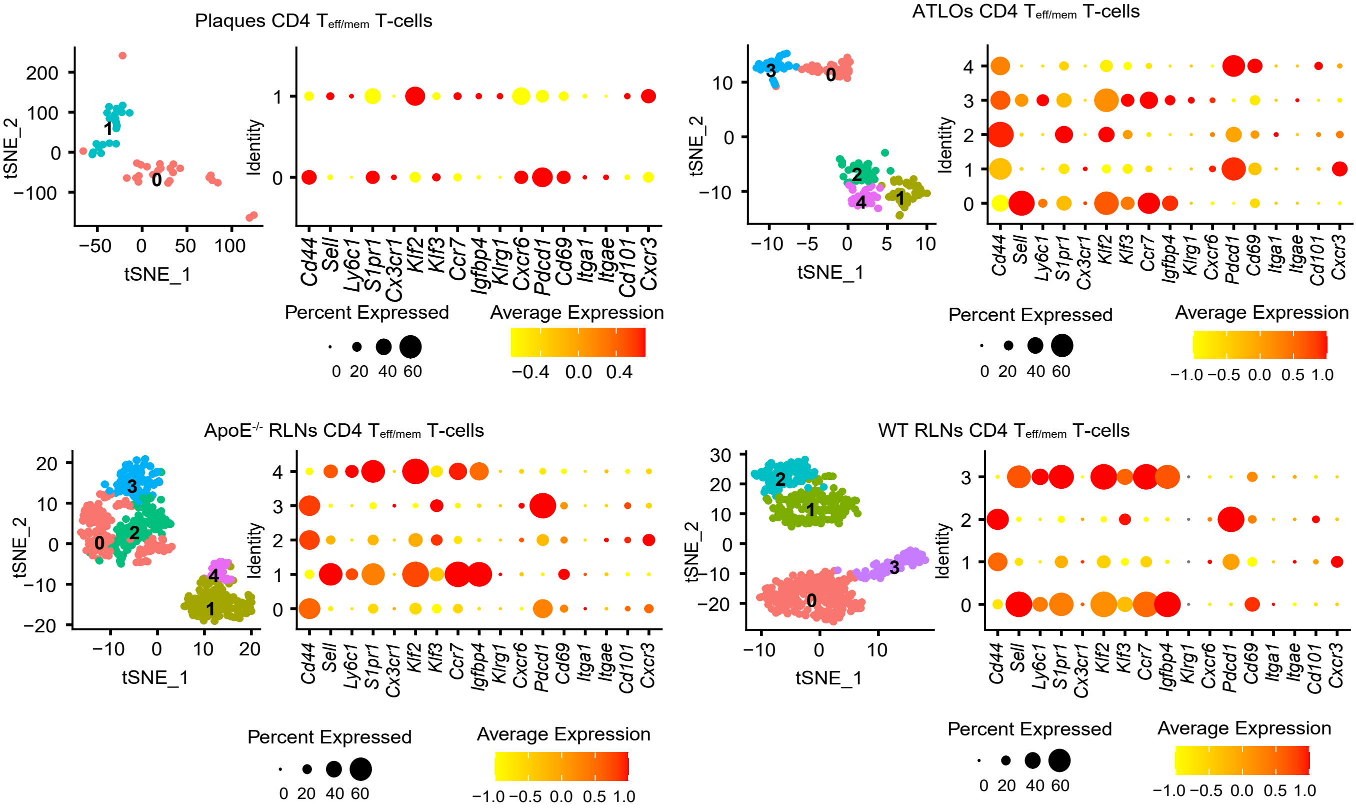

Supplementary Fig. 1: Average expression of genes with similarities to tissue-resident memory (TRM)-like T-cells in different CD4 $T_{eff/mem}$ T-cells among different tissues.
